# Structural insights into the function of ZRANB3 in replication stress response

**DOI:** 10.1038/ncomms15847

**Published:** 2017-06-16

**Authors:** Marek Sebesta, Christopher D. O. Cooper, Antonio Ariza, Christopher J. Carnie, Dragana Ahel

**Affiliations:** 1 Sir William Dunn School of Pathology, University of Oxford, Oxford OX1 3RE, UK

## Abstract

Strategies to resolve replication blocks are critical for the maintenance of genome stability. Among the factors implicated in the replication stress response is the ATP-dependent endonuclease ZRANB3. Here, we present the structure of the ZRANB3 HNH (His-Asn-His) endonuclease domain and provide a detailed analysis of its activity. We further define PCNA as a key regulator of ZRANB3 function, which recruits ZRANB3 to stalled replication forks and stimulates its endonuclease activity. Finally, we present the co-crystal structures of PCNA with two specific motifs in ZRANB3: the PIP box and the APIM motif. Our data provide important structural insights into the PCNA-APIM interaction, and reveal unexpected similarities between the PIP box and the APIM motif. We propose that PCNA and ATP-dependency serve as a multi-layered regulatory mechanism that modulates ZRANB3 activity at replication forks. Importantly, our findings allow us to interpret the functional significance of cancer associated *ZRANB3* mutations.

## Abstract (web summary)

ZRANB3 (Zinc-finger, RAN-Binding domain containing 3) is a structure-specific endonuclease that is recruited to DNA breaks and stressed replication forks. Here the authors present the crystal structure of the ZRANB3 endonuclease domain and analyse how ZRANB3 is regulated by the DNA clamp PCNA.

DNA replication is a fundamental process critical for cell survival. To achieve successful transmission of genetic information, cells must ensure precise and timely duplication of their genomic content. This is compounded by the fact that specific conditions impose ‘replication stress’ and interfere with the replication process. Fortunately, cells have evolved mechanisms capable of dealing with a wide range of genotoxic insults.

Cellular responses to replication-associated DNA damage are centred around Proliferating Cell Nuclear Antigen (PCNA), a DNA clamp that acts as a processivity factor for eukaryotic DNA polymerases^1^. The roles of PCNA in normal DNA replication and in the replication stress response are supported by its ability to act as a molecular platform for the recruitment of specific protein factors. The interactions of PCNA with its protein partners are mediated through specific PCNA interaction peptides, which generally belong to one of two types. One is the PCNA-Interacting Protein (PIP) box, a short sequence defined by Q-x-x-[VILM]-x-x-[FY]-[FY] consensus^2^, and the other is the AlkB homologue 2 PCNA Interacting Motif (APIM), characterized by the sequence [KR]-[FYW]-[LIVA]-[LIVA]-[KR]^3^. Available co-crystal structures reveal that PIP box peptides interact with the hydrophobic pocket on the PCNA surface, buried under the long stretch of residues known as the interdomain connecting loop (IDCL)^1^. However, in contrast to the multitude of structures available for different PCNA:PIP box complexes, an atomic resolution structure of PCNA:APIM complex is currently missing.

Among the proteins that interact with PCNA are structure-specific nucleases associated with DNA replication and DNA repair, such as Zinc-finger, RAN-Binding domain containing 3 (ZRANB3) and Flap Endonuclease 1 (FEN1). In general, structure-specific nucleases process secondary DNA structures that arise as intermediates during DNA replication, DNA repair and recombination. FEN1 processes the 5′-flap structures in DNA replication and DNA repair^4^. Roles of FEN1 in numerous DNA metabolic pathways have been studied intensely over the years and our insight into its function is supported by the available structural data^5^,^6^,^7^,^8^. In contrast to this, ZRANB3 is relatively underexplored and its biological roles are not well understood.

Previous studies have shown that ZRANB3 localizes to the sites of ongoing DNA replication, and interacts with PCNA and subunits of the replicative helicase complex MCM^9^. Furthermore, it is recruited to DNA breaks and stressed replication forks, and its deficiency leads to an increased sensitivity to certain DNA damaging agents^9^,^10^,^11^. Structurally, ZRANB3 belongs to the Sucrose Non-Fermenting 2 (SNF2) family of ATPases and contains several identifiable domains and motifs, which provide specificity for a number of defined substrates. These include the amino-terminal SNF2 ATPase core, two PCNA binding motifs (the PIP box and the APIM motif), a specific type of zinc-finger (Npl4 Zinc-Finger, NZF) and the HNH nuclease domain (named after the three most conserved residues) [Fig f1][Fig f2][Fig f3](Fig. 4b).

Unlike FEN1, ZRANB3 is a highly unusual endonuclease and cleaves its DNA substrate in an ATP-dependent manner^9^. The nuclease activity is supported by the carboxy-terminal HNH domain, which is also found in a variety of bacterial and viral proteins (including homing endonucleases, bacterial toxins, restriction endonucleases, transposases and DNA packaging factors^12^,^13^). Bacterial HNH domains are known to act as both site-specific DNA nucleases (in homing nucleases for example, or in the RNA-guided DNA endonuclease Cas9), as well as non-specific nucleases (in colicins, a subgroup of bacterial toxins)^14^,^15^. Interestingly, in eukaryotic ZRANB3 protein the HNH domain supports the structure-specific endonuclease activity^9^, which might be associated with the role of ZRANB3 in the replication stress response. However, it remains unclear how this is achieved on a molecular basis.

Here, we present a detailed analysis of ZRANB3 activity and provide structural insights into its endonuclease function. We also explore the relevance of the ZRANB3:PCNA interaction and consider the biological implications of this association. Our data highlight multiple roles of PCNA, both as a molecular scaffold for the recruitment of replication-associated protein complexes, as well as a specific modulator of enzymatic activities. We show that PCNA not only plays a critical role in targeting ZRANB3 to the sites of DNA replication, but that it also serves as a cofactor and stimulates ATP-dependent ZRANB3 endonuclease activity. Furthermore, we provide structural evidence of the PCNA:APIM complex and demonstrate that, despite the differences in amino-acid sequence, the PIP box and the APIM motif share similar topology and bind to the same molecular surface of the PCNA ring. Finally, our structural and biochemical analyses enable us to assess the functional relevance of cancer associated mutations of *ZRANB3*. We demonstrate a high incidence of impaired and/or loss-of-function mutations that likely contribute to genomic instability in this context.

## Results

### Structure of the ZRANB3 HNH domain

**Figure 1 f1:**
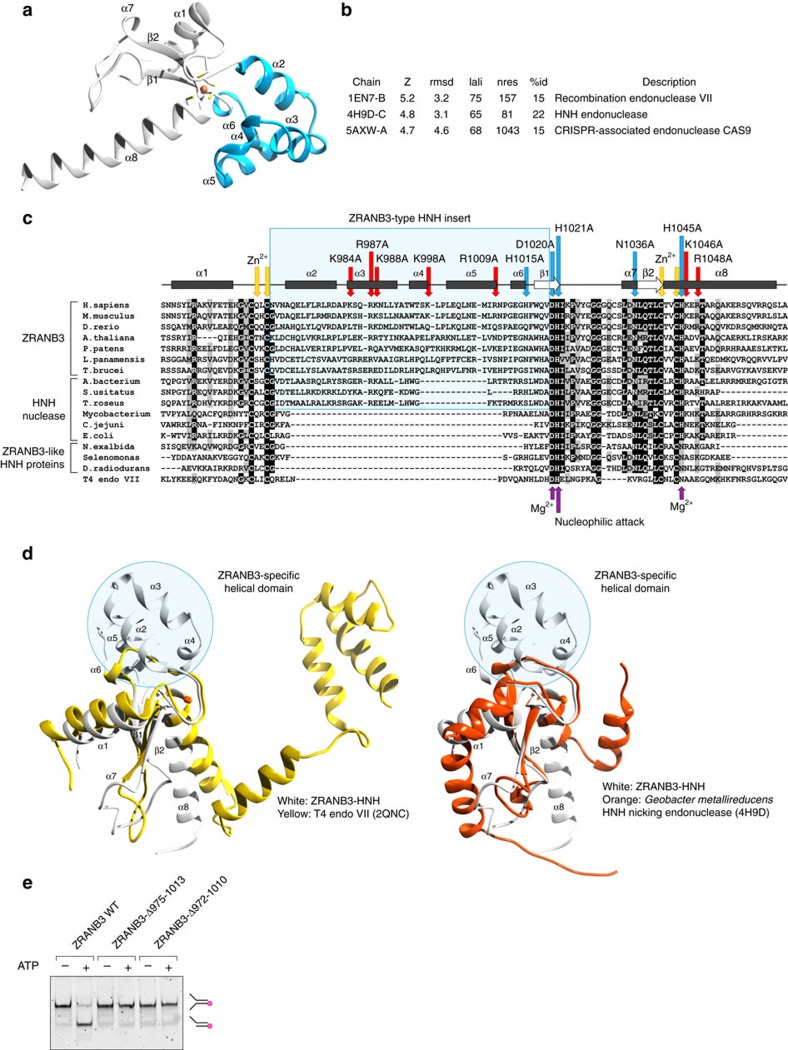
Crystal structure of the ZRANB3 HNH domain. (**a**) Crystal structure of the ZRANB3 HNH domain (residues 948–1,067). Secondary structure elements are annotated (helices α1–α8 and β-strands β1–β2). A bound zinc ion is shown as an orange sphere, with its four coordinating cysteine residues shown as sticks. The ZRANB3-specific helical domain is shown in blue. (**b**) Top scoring structural homologues of the ZRANB3 HNH domain identified by DALI search^17^. (**c**) Sequence alignment of the HNH domains from eukaryotic ZRANB3 proteins, bacterial self-standing HNH nucleases and bacterial ZRANB3-like proteins. Secondary structure elements are shown for human ZRANB3 HNH domain only. Targeted mutation sites are indicated by red (basic residues) and blue (active site residues) arrows; residues involved in zinc ion coordination are indicated by yellow arrows; T4 endonuclease VII active site residues are indicated by purple arrows. (**d**) Overlay of the ZRANB3 HNH (white) with the structures of T4 endonuclease VII (yellow) and *Geobacter metallireducens* HNH nicking endonuclease (orange). Non-catalytic zinc ions are shown as orange spheres. (**e**) Nuclease assay with full-length ZRANB3 and the two mutants containing deletions of the ZRANB3-specific helical domain (ZRANB3-Δ975–1013 and ZRANB3-Δ972–1010). DNA substrate (splayed DNA duplex fluorescently labelled at the 5′ end) and the resulting product of the nucleolytic cleavage (fluorescently labelled DNA duplex with 5′ overhang) are indicated in the picture. Shown are reactions in the presence and absence of ATP. Reactions were analysed by native polyacrylamide gel electrophoresis.

To gain structural insight into the ZRANB3 HNH domain, we solved the crystal structure of the C-terminal region of the ZRANB3 (residues 948-1067) by experimental phasing. The HNH structure contains two HNH molecules in the asymmetric unit that are structurally similar, with an RMSD of 0.76 Å over 117 equivalent Cα atoms[Table t1]. Each HNH molecule contains one two-stranded anti-parallel β-sheet (β1–β2) surrounded by eight helices (α1–α8). The structure adopts a ββα fold^16^, typically found in the HNH protein family. It also contains a non-catalytic zinc-finger coordinated with four cysteine residues (Fig. 1a and Supplementary Fig. 1), conserved in a subset of HNH proteins containing the C-x-x-C dyad^14^. A structural homology search using the DALI server^17^ identified the closest structural homologues of the ZRANB3 HNH domain as phage T4 endonuclease VII, a Holliday junction-specific resolvase (PDB code 1EN7; ref. 18), and the HNH nicking endonuclease from *Geobacter metallireducens* (PDB code 4H9D; ref. 19) (Fig. 1b). Interestingly, sequence comparisons with other zinc-finger containing HNH proteins revealed the presence of the HNH insert (Fig. 1c), specific to ZRANB3 and the self-standing HNH proteins found in *Acidobacterium bacterium* and *Solibacter usitatus*, but absent from other HNH proteins. Overlays of the ZRANB3 HNH structure with its closest structural homologues showed that this ZRANB3-specific HNH insert adopts a well-structured α-helical domain (Fig. 1a,d). Deletion of this domain led to the loss of ZRANB3 endonuclease activity (and a reduction, but not a complete loss of the ATPase activity, Fig. 1e and Supplementary Fig. 2a; Table 2), raising a possibility that it might be important for the structure-specific recognition of the DNA substrate.

### DNA binding surface

**Figure 2 f2:**
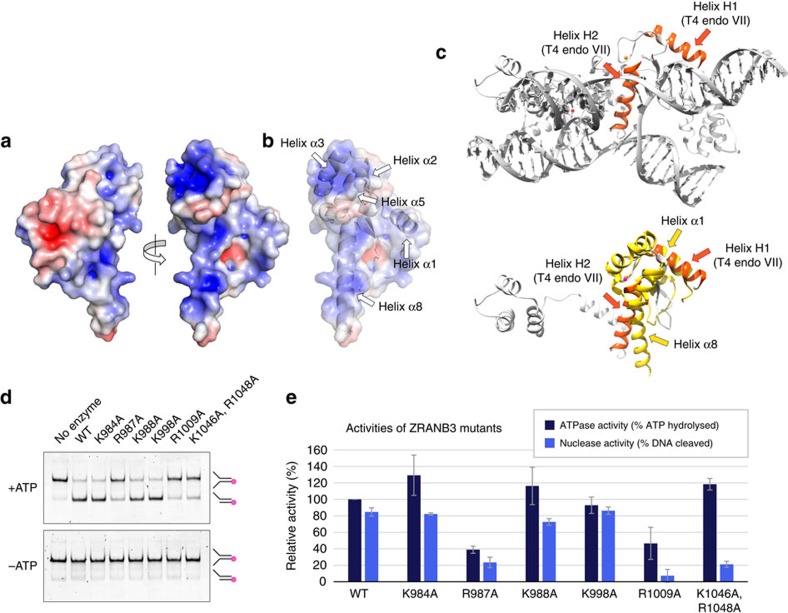
DNA binding surface. (**a**) Electrostatic surface potential (blue, positive; red, negative) of the ZRANB3 HNH domain. (**b**) Electrostatic surface potential with indicated secondary structure elements. (**c**) Comparison of the T4 endonuclease VII and ZRANB3 HNH domains. H2 helices in T4 endonuclease VII stabilize separation between DNA strands in the Holliday junction (top). Helices H1 and H2 in T4 endonuclease VII (orange; only one subunit shown for clarity) align with helices α1 and α8 in the ZRANB3 HNH structure (yellow) (bottom). (**d**) Nuclease assay with the wild-type and mutant ZRANB3 proteins in the presence and absence of ATP. Reactions were analysed by native polyacrylamide gel electrophoresis. Indicated are mobilities of the fluorescently labelled DNA substrate and product. (**e**) Quantification of the ATPase and nuclease activities of the wild-type and mutant ZRANB3 proteins. Shown are the averages of four ATPase and five nuclease reactions. s.d.’s are shown as error bars.

Analysis of the electrostatic surface potential of the HNH domain revealed a predominantly electropositive face (Fig. 2a), with a continuous positively charged region encompassing helices α1 and α8, as well as helices α2 and α3 in the ZRANB3-specific helical domain (Fig. 2b). Overlay of the HNH and T4 endonuclease VII structures showed that helix H2 in T4 endonuclease VII, which penetrates into the Holliday junction and stabilizes the separation between exchanging DNA strands, overlaps with the helix α8 in the HNH structure (Fig. 2c), suggesting a potential involvement of helix α8 in DNA binding. To address the role of the positively charged regions of the HNH domain, we introduced a number of mutations targeting the ZRANB3-specific helical region and helix α8 (Fig. 1c). Some of these mutations resulted in reduced DNA binding by the HNH domain (Supplementary Fig. 3b), and had a pronounced effect on the ZRANB3 endonuclease activity- as observed with the K1046A, R1048A double mutant in helix α8, and with the R1009A mutant in the ZRANB3-specific helical domain (Fig. 2d,e). Interestingly, the K1046A, R1048A double mutant retained efficient ATPase activity (Fig. 2e, Table 2), suggesting that the impact of the mutations on the endonuclease function was not linked to the ability to hydrolyse ATP. The DNA binding surface therefore likely involves helix α8 and certain positively charged regions within the ZRANB3-specific helical domain.

### Characterization of the active site

**Figure 3 f3:**
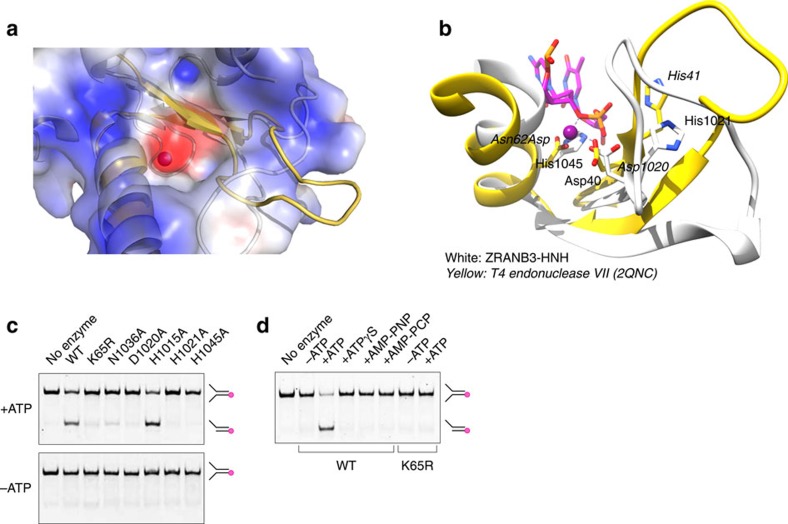
Characterisation of the ZRANB3 HNH active site. (**a**) Overlay of the T4 endonuclease VII (yellow) and the ZRANB3 HNH (white) structures. Electrostatic surface potential of the HNH domain shows an overlap of the electronegative patch (red) with the Mg^2+^ ion (purple) in the T4 endonuclease VII active site. (**b**) Overlay of the T4 endonuclease VII (yellow, labels in italics) and the ZRANB3 HNH (white) structures. Mg^2+^ ion in T4 endonuclease is shown as a purple sphere. His1021, Asp1020 and His1045 in the HNH structure overlap with the active site residues of T4 endonuclease VII. (**c**) Nuclease assay with the wild-type and mutant ZRANB3 proteins in the presence and absence of ATP. Reactions were analysed by native poly-acrylamide gel electrophoresis. Indicated are mobilities of the fluorescently labelled DNA substrate and product. (**d**) Nuclease assay with non-hydrolysable ATP homologues. Shown are controls with the wild-type ZRANB3 and the ATPase dead K65R mutant in the presence of ATP.

Analysis of the electrostatic surface potential also revealed the presence of a negatively charged patch on a predominantly electropositive face of the HNH molecule (Figs 2a and 3a). Interestingly, overlay of the HNH and T4 endonuclease VII structures showed that the observed electronegative patch in HNH overlaps with the Mg^2+^ ion binding pocket in the T4 endonuclease VII active site (Fig. 3a). To gain insight into the architecture of the HNH catalytic site, we further analysed the structure of the ZRANB3 HNH domain superimposed to that of the inactive T4 endonuclease VII (N62D) in complex with the Holliday junction DNA substrate. The active site of T4 endonuclease VII adopts a characteristic ββα-metal fold, and contains a Mg^2+^ ion and catalytically essential residues Asp40, His41 and Asn62 (refs 20, 21, 22) (Fig. 3b). The Mg^2+^ ion is coordinated by the side chains of Asp40 and Asn62Asp, two oxygen atoms of the scissile phosphate and a water molecule^22^. A catalytic mechanism involves His41, which likely acts as a general base and activates water for nucleophilic attack. On the other hand, the Mg^2+^ ion is thought to stabilize the phosphoanion transition state and facilitate product formation. Although the structure of the ZRANB3 HNH domain does not reveal the presence of a divalent cation in the putative active site, residues Asp1020, His1021 and His1045 assume equivalent positions to the catalytically important residues within the ββα-metal fold of T4 endonuclease VII (Fig. 3b). Sequence comparisons show strict conservation of Asp1020 and His1021 and His1045 among ZRANB3 proteins, highlighting their importance (Fig. 1c). By analogy with the T4 endonuclease VII structure, we hypothesized the involvement of Asp1020 and His1045 in metal coordination, and the role of His1021 as a general base in nucleophilic attack.

To address the relevance of specific residues in the ββα-fold of HNH domain, we introduced a number of mutations and tested the activities of the mutant ZRANB3 proteins. Although the mutations did not have a dramatic effect on DNA-dependent ATP hydrolysis (Supplementary Fig. 2c, Table 2), some of them either substantially reduced (N1036A), or completely abrogated (D1020A, H1021A and H1045A) ZRANB3 endonuclease activity (Fig. 3c). This is consistent with the proposed catalytic roles of Asp1020, His1021 and His1045 in the nucleolytic cleavage of DNA substrate.

ZRANB3 differs from other characterized HNH nucleases in being an ATP-dependent nuclease. Our previous work showed that mutation of the conserved Lys65 in the ATP-binding motif of ZRANB3 (known as the Walker A motif) yielded an ATPase-deficient enzyme^9^. Although the ATPase dead ZRANB3 mutant K65R showed no detectable endonuclease activity, it was unclear whether this was due to its inability to hydrolyse ATP, or due to the possible effect of the K65R mutation on the binding of ATP. To test whether ATP hydrolysis is essential for endonuclease activity, we tested ZRANB3 endonuclease activity in the presence of non-hydrolysable ATP analogues (ATPγS, AMP-PNP, AMP-PCP). Our data show that substitution of ATP by non-hydrolysable ATP analogues in ZRANB3 endonuclease reactions resulted in a complete loss of activity (Fig. 3d). ZRANB3 endonuclease activity is therefore strictly dependent on ATP hydrolysis catalysed by the helicase core domain.

### Stimulation of nuclease activity by PCNA

**Figure 4:**
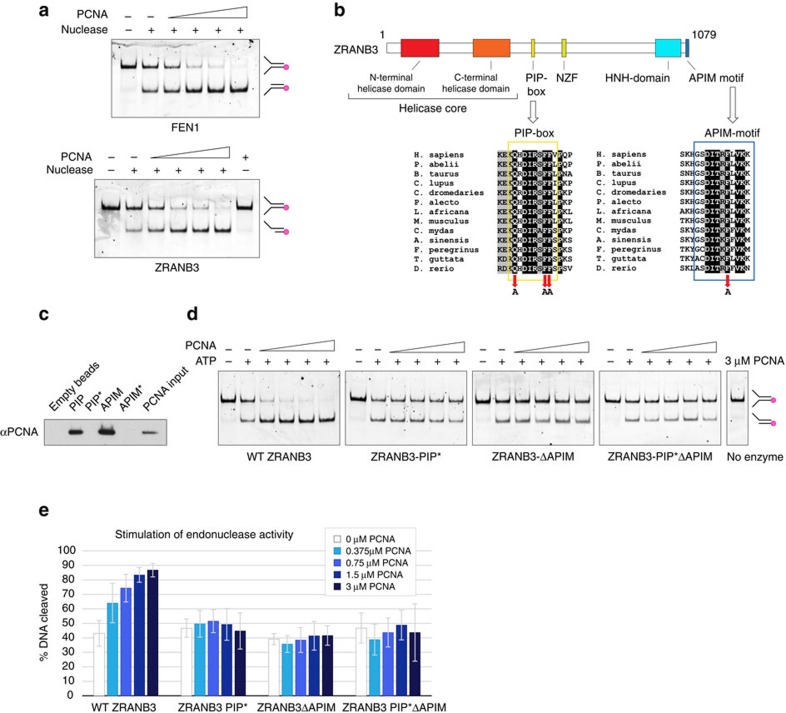
Stimulation of the ZRANB3 nuclease activity by PCNA. (**a**) Stimulation of FEN1 and ZRANB3 nuclease activities by PCNA. FEN1 or ZRANB3 nucleases were incubated with the DNA substrate and increasing concentrations of PCNA (0.375, 0.75, 1.5 and 3 μM), in the presence (ZRANB3) or absence (FEN1) of ATP. (**b**) Domain structure of ZRANB3. Conservation of the PIP box and the APIM motif residues among ZRANB3 proteins are shown in the alignments below. Mutated conserved residues in the PIP box and the APIM motif are indicated by arrows. (**c**) Interactions of the ZRANB3 PIP box and APIM motif with PCNA. Biotinylated PIP box and APIM motif peptides were bound to streptavidin beads and incubated with recombinant PCNA. The interaction was assayed by Western blotting with PCNA antibody. Mutations of the conserved residues (Q519A, F525A, and F526A in PIP* and F1075A in APIM*) abrogated the interaction with PCNA. (**d**) Effect of PCNA (0.375, 0.75, 1.5 and 3 μM) on ZRANB3 nuclease activity assayed with the wild-type ZRANB3 and ZRANB3 PCNA binding mutants (ZRANB3-PIP*, ZRANB3-ΔAPIM and ZRANB3-PIP*ΔAPIM). (**e**) Quantification of nuclease activities in the presence of increasing concentrations of PCNA. Shown are averages of five reactions. s.d.’s are shown as error bars.

Given that ZRANB3 acts as a structure-specific endonuclease, we compared its activity to an archetypal structure-specific endonuclease: FEN1. FEN1 is known to interact with PCNA through the PIP box located at its C-terminus^5^,^6^, and this association was shown to stimulate endonuclease activity of FEN1 (refs 23, 24). We therefore sought to test whether PCNA also has an effect on the endonuclease activity of ZRANB3. Our results show that, similarly to FEN1 activation, increasing concentrations of PCNA stimulated endonuclease activity of ZRANB3 (Fig. 4a). To gain molecular insight into this activation, we decided to explore the way ZRANB3 interacts with PCNA. Previous studies identified two conserved PCNA binding motifs in ZRANB3: a PIP box located between residues 519 and 526, and an APIM motif located at the very C-terminal end of the ZRANB3 protein (Fig. 4b, ref. 10). We inactivated the PIP box and the APIM motif by mutating critical residues (Fig. 4b,c), and assessed the impact of PCNA interactions on ZRANB3 endonuclease activity. Whereas the wild-type ZRANB3 showed notable stimulation of endonuclease activity in the presence of PCNA, such stimulation could not be observed upon inactivation of either the PIP box or the APIM motif (mutants PIP* and ΔAPIM, respectively) (Fig. 4d,e). An additive effect could not be observed with the PIP*-ΔAPIM double mutant, which highlights the relevance of both PCNA binding motifs for optimal ZRANB3 endonucleolytic activity. Interestingly, PCNA did not stimulate the ATPase activity of ZRANB3 (Supplementary Fig. 2d). Moreover, mutations of the PCNA binding motifs did not affect ATPase function (Supplementary Fig. 2e, Table 2), suggesting that ZRANB3 employs distinct mechanisms for activation (by ATP hydrolysis) and stimulation (by PCNA binding) of its endonuclease activity.

### PCNA-dependent recruitment of ZRANB3

**Figure 5:**
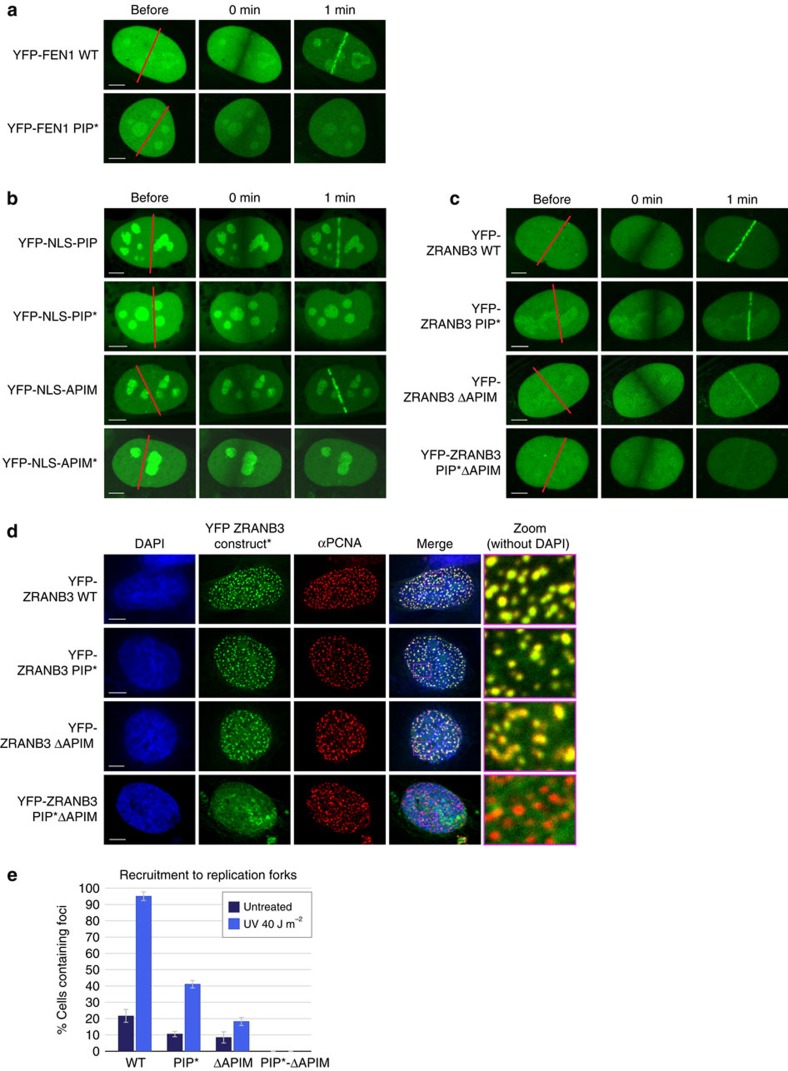
PCNA-dependent recruitment of ZRANB3. (**a**) Recruitment of FEN1 to the sites of laser induced DNA damage is abrogated by the mutations in the PIP box (Q337A, F343A and F344A). U2OS cells were transiently transfected with the indicated YFP constructs and analysed by live-cell imaging. Shown are representative images at the indicated time points post damage. (**b**) Recruitment of the YFP-tagged PIP box and APIM motif to the sites of laser induced DNA damage. Recruitment is abrogated by the mutations of the conserved residues in the PIP box and the APIM motif. (**c**) Recruitment of the YFP-tagged wild-type ZRANB3 and ZRANB3 PCNA binding mutants ZRANB3-PIP*, ZRANB3-ΔAPIM and ZRANB3-PIP*ΔAPIM) to the sites of laser induced DNA damage. (**d**) Recruitment of the wild-type and mutant ZRANB3 proteins to the sites of ongoing DNA replication. U2OS cells were transiently transfected with the indicated YFP constructs and stained against endogenous PCNA. Scale bar (**a**–**d**) 5 μm. (**e**) ZRANB3 accumulates at stalled replication forks. U2OS cells were transfected with YFP-ZRANB3 constructs and either left untreated, or exposed to UV irradiation. After 6 h, cells were fixed and stained with PCNA antibody. The percentage of cells containing ZRANB3 foci that colocalize with PCNA was determined. Shown is the average of three experiments. s.d.’s are shown as error bars.

We next wanted to address the importance of PCNA in targeting structure-specific nucleases to sites of DNA damage. We expressed YFP-tagged proteins in U2OS cells and examined their ability to localize at sites of DNA damage caused by Ultraviolet laser microirradiation. As shown in Fig. 5a, YFP-FEN1 was efficiently recruited to the sites of laser-induced DNA damage. However, mutation of the PIP box motif resulted in a dramatic loss of FEN1 recruitment to the microirradiated stripes (YFP-FEN1 PIP* mutant), highlighting the importance of PCNA in mobilizing FEN1 to the appropriate nuclear locations. We further examined the recruitment of ZRANB3 to DNA damage, which was previously suggested to employ a similar PCNA-dependent mechanism^9^,^10^. To address the relevance of the individual PCNA binding motifs in ZRANB3 more directly, we expressed them as YFP-tagged proteins with nuclear localization signals. As shown in Fig. 5b, both the PIP box and the APIM motif were efficiently mobilized to the sites of DNA damage. However, this was completely abrogated when mutations, shown to abolish interactions with PCNA (Fig. 4c), were introduced into the PIP box and the APIM motif (Fig. 5b, mutants PIP*and APIM*). To address the relevance of these motifs in the context of the full length ZRANB3 protein, we further tested the recruitment of YFP-ZRANB3 wild-type and mutant proteins to locally induced DNA damage. In agreement with the published data^10^, ZRANB3 was readily recruited to the microirradiated stripes, and the individual inactivation of either the PIP box or the APIM motif (mutants PIP* and ΔAPIM, respectively) was not sufficient to abrogate recruitment to DNA damage (Fig. 5c). The recruitment of ZRANB3 was abolished only upon inactivation of both PCNA binding motifs (PIP*-ΔAPIM double mutant, Fig. 5c), indicating that the PIP box and the APIM motif might act independently in a biological context to support PCNA-dependent recruitment to DNA damage.

We also examined the relevance of PCNA binding motifs in targeting ZRANB3 to the sites of DNA replication. To do this, we expressed YFP-ZRANB3 wild-type and mutant proteins and analysed their ability to colocalize with endogenous PCNA. In undamaged conditions, the majority of YFP-ZRANB3 expressing cells show uniform nuclear expression (such as in Fig. 5c). However, about 20% of the cells expressing YFP-ZRANB3 form foci, which colocalize with PCNA (Fig. 5d,e and Supplementary Fig. 4) (ref. 9). Individual mutation of either of the PCNA binding motifs in ZRANB3 reduced the efficiency of foci formation (PIP* and ΔAPIM mutants, Fig. 5e), but a complete loss of colocalization with PCNA was observed only upon inactivation of both motifs (PIP*-ΔAPIM double mutant, Fig. 5d,e). This indicates that the PIP box and the APIM motif mediated interactions with PCNA play critical roles in the recruitment of ZRANB3 to the sites of ongoing DNA replication.

ZRANB3 is also known to play a role in the replication stress response and to accumulate at sites of stalled DNA replication^9^,^10^,^11^. To address the significance of PCNA-binding motifs in recruiting ZRANB3 to stalled replication forks, we expressed YFP-ZRANB3 wild-type and mutant proteins in U2OS cells, and induced replication stress by exposing the cells to ultraviolet light (UV) irradiation. Following a 6-h recovery, we evaluated recruitment of ZRANB3 to stressed replication forks by measuring colocalization of YFP-ZRANB3 proteins with endogenous PCNA. UV induced an increase in the number of cells that formed ZRANB3 foci, and for wild-type ZRANB3 the proportion changed from ∼20% of cells in undamaged conditions to >95% following Ultraviolet irradiation (Fig. 5e and Supplementary Fig. 5). The number of cells containing focally concentrated ZRANB3 also increased with the PIP* and ΔAPIM mutants following Ultraviolet irradiation, but not with the PIP*-ΔAPIM double mutant, whose ability to form foci was completely lost (Fig. 5e). Interestingly, the increase in the number of cells containing ZRANB3 foci was more pronounced with the PIP* mutant than with the ΔAPIM mutant, possibly suggesting a specific role of the APIM motif in the replication stress response.

### Comparative analysis of PCNA binding affinities

We further examined the association of PCNA and the peptides derived from ZRANB3 by performing isothermal titration calorimetry (ITC) (Supplementary Fig. 6). Analyses of the synthetic peptides containing the PIP box or APIM motif sequences revealed comparable affinities of the two peptides for PCNA (*K*_D_ values of 4.8 and 9.24 μM for the PIP box and the APIM motif, respectively) (Supplementary Table 1). In agreement with previous studies, the association of the peptides with PCNA fitted a 1:1 stoichiometry, suggesting that the trimeric complex can accommodate one peptide per monomer^5^,^25^,^26^,^27^. Furthermore, the associations of ZRANB3 peptides were compared to the interactions of peptides derived from other PCNA interacting proteins (FEN1, p21 and Polι). The peptides were of equal length and covered the homologous sequence within the PIP box region. Our data showed that the affinities of ZRANB3 peptides were within the range of values detected for other PIP box peptides (Supplementary Table 1).

### Structures of the PCNA-PIP and PCNA-APIM complexes

To gain molecular insight into the way the PIP box and the APIM motif of ZRANB3 interact with PCNA, we crystallized and determined the co-structures of PCNA with the ZRANB3 PIP and APIM peptides (Table 1). Both the PCNA:ZRANB3(PIP) and PCNA:ZRANB3(APIM) complexes show the typical homo-trimeric PCNA structure, consisting of three PCNA molecules in the asymmetric unit forming a trimeric ring^28^. Three peptide molecules are visible in the structures, each of which interacts with a different monomer in the PCNA trimer.

**Figure 6:**
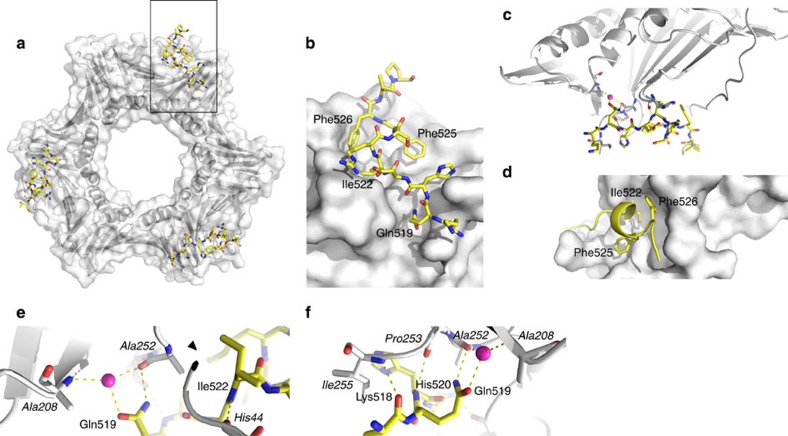
Structure of the PCNA:ZRANB3(PIP) complex. (**a**) Front view of the PCNA ring (grey surface and ribbons) with the ZRANB3 PIP box peptide (yellow sticks). (**b**) Magnified view of the boxed region in **a**. Shown is a surface representation of one of the three PIP box binding sites on the PCNA ring with the bound PIP box peptide (yellow sticks coloured by atom type). (**c**) Overview of the hydrogen-bond interaction network between the ZRANB3 PIP box peptide (yellow) and PCNA (grey). Bound water molecule (purple sphere) and hydrogen bonds (yellow dotted lines) are shown. (**d**) Hydrophobic pocket on PCNA surface (grey) with conserved residues that form ‘hydrophobic plug’(Ile 522, Phe525 and Phe526; shown as yellow sticks) in the ZRANB3 PIP box peptide. (**e**,**f**) Magnified view of the hydrogen-bond interaction network between the ZRANB3 PIP box peptide (yellow) and PCNA (grey, labels in italics). Bound water molecule (purple sphere) and hydrogen bonds (yellow dotted lines) are shown.

**Figure 7:**
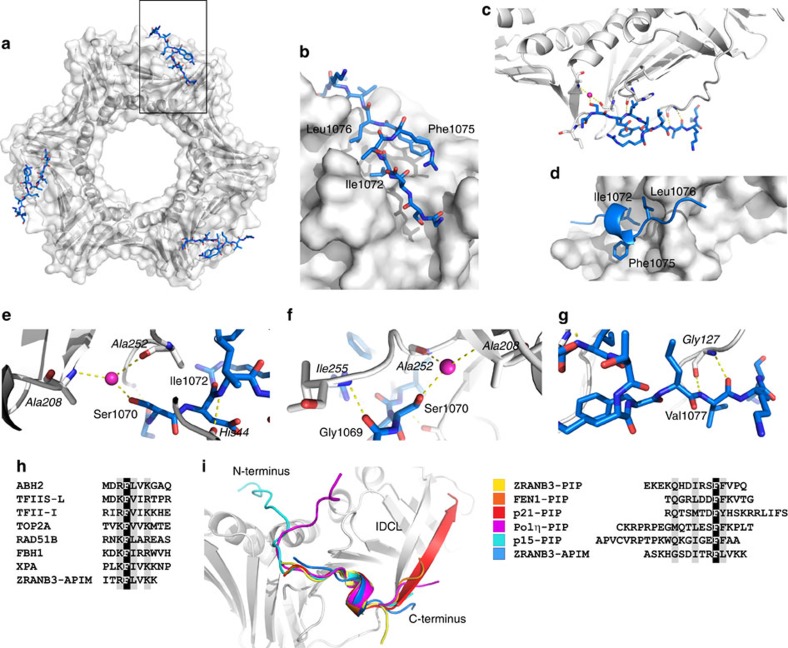
Structure of the PCNA:ZRANB3(APIM) complex. (**a**) Front view of the PCNA ring (grey surface and ribbons) with the ZRANB3 APIM motif peptide (blue sticks). (**b**) Magnified view of the boxed region in **e**. Shown is a surface representation of one of the three APIM motif binding sites on the PCNA ring with the bound APIM motif peptide (blue sticks coloured by atom type). (**c**) Overview of the hydrogen-bond interaction network between the ZRANB3 APIM motif peptide (blue) and PCNA (grey). Bound water molecule (purple sphere) and hydrogen bonds (yellow dotted lines) are shown. (**d**) Hydrophobic pocket on PCNA surface (grey) with the residues that form ‘hydrophobic plug’ in the APIM motif (Ile 1072, Phe1075 and Leu1076; shown as blue sticks). (**e**–**g**) Magnified view of the hydrogen-bond interaction network between the ZRANB3 APIM motif peptide (blue) and PCNA (grey, labels in italics). Bound water molecule (purple sphere) and hydrogen bonds (yellow dotted lines) are shown. (**h**) Alignment of the APIM motifs from different human proteins. (**i**) Superimposed backbones of bound PIP box and APIM motif peptides from different co-crystal structures. Alignment of the peptides is shown on the right. Shown are ZRANB3-PIP^515–529^ (yellow; PBD:5MLO), FEN1^336–348^ (orange; PDB:1U7B), p21^143–160^ (red; PDB: 1AXC), Polη^694–713^ (magenta; PDB: 2ZVK), p15^51–71^ (cyan; PDB: 4D2G) and ZRANB3-APIM^1065–1079^ (blue; PDB:5MLW).

The PIP boxes found in PCNA interaction partners can generally be categorized as either canonical (defined by the Q-x-x-[ILM]-x-x-F-[FY] consensus sequence) or non-canonical (deviate from the canonical consensus). ZRANB3 has a canonical PIP box (Fig. 4b) that binds to the PCNA ring in a manner similar to other canonical PIP boxes (such as those found in p66 of Polδ and FEN1; Figs 6a–f and 7i)^5^. Specifically, the conserved glutamine (Gln519) in the PIP box peptide interacts with PCNA by forming a hydrogen bond with the main-chain carbonyl of Ala252, and by an additional contact with Ala208 via a well-structured water molecule (Fig. 6e,f). Furthermore, Lys518 and His520 form main chain hydrogen bonds with Ile255 and Pro253 of PCNA, respectively (Fig. 6e,f and Supplementary Fig. 8a). On the other hand, residues Ile522-Phe526 form a 3_10_ helix that orients the conserved hydrophobic residues (also known as the ‘hydrophobic plug’^1^; Ile522, Phe525 and Phe526 in the ZRANB3 PIP box) for docking into the hydrophobic patch on the PCNA surface (Fig. 6d). While this topology is conserved in other PIP box peptides, differences are usually observed in the regions at the C-terminus of the PIP box sequence. This region forms an antiparallel β-sheet with the IDCL of PCNA in p21 (ref. 25) and FEN1 (ref. 6), but not in some other proteins with canonical PIP boxes, such as the PIP box in the p66 subunit of Polδ^5^ or the PIP box in the intrinsically disordered protein p15(PAF)^29^ (Fig. 7i). In the structure presented here, the PIP box peptide is not long enough to form extensive contacts with the IDCL.

Although the APIM motif has been characterized as a PCNA-binding motif in a number of proteins^3^ (Fig. 7h), the structural insight into its mode of interaction with PCNA is still lacking. Our structure of PCNA:ZRANB3(APIM) shows that the APIM motif occupies the same general binding site on the PCNA surface as do canonical and non-canonical PIP boxes (Fig. 7a–c,i and Supplementary Fig. 8c)^30^. Moreover, the basic topology of the PIP box defined by a 3_10_ helix is conserved in the APIM motif (Fig. 7d), despite the apparent differences in the amino-acid sequence. The 3_10_ helical conformation is stabilized by a network of intramolecular hydrogen bonds; however, whereas in PIP boxes this network does not seem to necessitate conservation of the constituent residues, in the APIM motif it involves a conserved basic residue (Arg1074 in ZRANB3; Fig. 7h), whose side chain nitrogen interacts with the backbone carbonyl of Asp1071. Moreover, Ile1072, Phe1075 and Leu1076 of the APIM motif form a hydrophobic plug, analogously to the conserved hydrophobic residues of the PIP box [ILM]-x-x-F-[FY] sequence (Fig. 7d,i). As in the PIP box structure, the 3_10_ helical configuration appropriately positions these residues into the hydrophobic patch on the PCNA surface.

Despite these similarities, the APIM motif interacts with PCNA in a specific way, which differs from previously characterized interactions of PCNA-interacting peptides. The most notable difference is the absence of a glutamine residue otherwise conserved in canonical PIP boxes (Fig. 7i), whose side chain forms both direct and water mediated hydrogen bonds with PCNA^25^,^29^ (Fig. 6e). The APIM motif contains a glycine at the analogous position (Gly1069), and is therefore unable to interact with PCNA in the same manner; instead, it forms a main chain–main chain hydrogen bond with Ile255 (Fig. 7f and Supplementary Fig. 8b). In addition, the side chain hydroxyl group of Ser1070 forms water mediated hydrogen bonds with Ala252 and Ala208, while Ile1072 forms a main chain hydrogen bond with His44 (Fig. 7e and Supplementary Fig. 8b). Differences are also observed in the formation of hydrophobic interactions: Leu1076 of the tripartite hydrophobic plug in the APIM motif has a smaller side chain than the corresponding phenylalanine of the PIP box peptide, and does not intrude as deeply into the PCNA binding pocket. This is not dissimilar to the non-canonical PIP box interaction of Polι with PCNA, which also contains a leucine residue at the corresponding position^31^.

The differences in the way the PIP box and the APIM motif of ZRANB3 bind to PCNA extend beyond the hydrophobic pocket on the PCNA surface. The C-terminal region of the APIM motif interacts with the IDCL, providing an additional point of contact. Specifically, the Val1077 of the APIM motif forms main-chain hydrogen bonds with Gly127 of PCNA (Fig. 7g). Interestingly, the corresponding region of Polη interacts with PCNA in a similar fashion and binds the same IDCL residue through equivalently positioned Lys709 (ref. 31). This differs from the extensive antiparallel β-sheets observed in the p21 and FEN1 PCNA complexes, which involve multiple intramolecular contacts^25^,^29^.

### Biological implications of ZRANB3 structural studies

**Figure 8:**
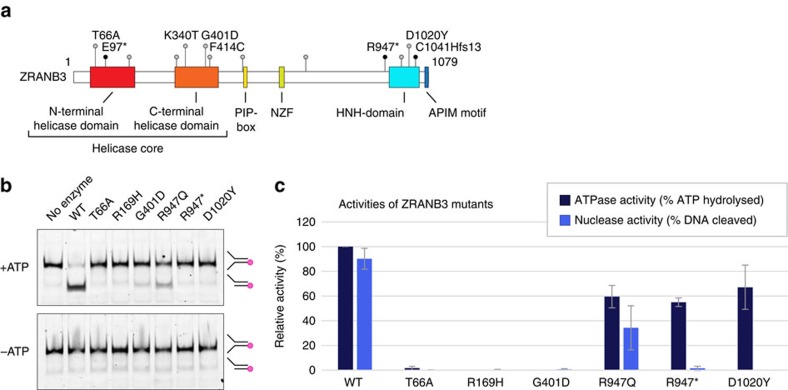
Cancer associated *ZRANB3* mutations. (**a**) *ZRANB3* mutations associated with endometrial carcinomas. Shown are positions of the mutations targeting conserved residues. Truncating mutations are indicated as black dots. (**b**) Nuclease assay with the wild-type and cancer associated mutant ZRANB3 proteins, in the presence and absence of ATP. Reactions were analysed by native polyacrylamide gel electrophoresis. Indicated are mobilities of the fluorescently labelled DNA substrate and product. (**c**) Quantification of the ATPase and nuclease activities of the wild-type and mutant ZRANB3 proteins. Shown are the averages of three ATPase and five nuclease reactions. s.d.’s are shown as error bars.

Replication stress is one of the major factors that contributes to genomic instability associated with the development of human cancers^32^,^33^,^34^. Although the role of ZRANB3 in the replication stress response has been evidenced by a number of studies^9^,^10^,^11^,^35^, pathobiological importance of its function has not been investigated. Interestingly, multiple *ZRANB3* variants have been identified in various human cancers, including endometrial carcinomas, a malignancy characterized by marked genomic instability^36^,^37^. Mapping the endometrial cancer associated *ZRANB3* variants onto the primary sequence of *ZRANB3*, we noted that the majority localized to functionally relevant domains, particularly the helicase core and the HNH domain^38^,^39^ (Fig. 8a). Furthermore, bioinformatic analyses (largely based on evolutionary conservation of the affected amino acids and on structural properties) suggested high probabilities of pathogenicity for majority of the *ZRANB3* variants Supplementary Table 2.

Three of the variants are predicted to yield truncated products (nonsense mutations E97*, R947* and a frameshift mutation C1041Hfs*13, Supplementary Table 3). Among these, E97* is expected to produce a functionally null allele, whereas R947* and C1041Hfs*13 are both expected to yield endonuclease deficient ZRANB3. Intriguingly, we noted an elevated frequency of highly conserved ZRANB3 residues among the mutations reported in the study (T66A, K340T, G401D, F414C and D1020Y), among which T66A and D1020Y are predicted to yield enzymatically inactive ZRANB3. Specifically, Thr66 is a conserved residue of a common nucleotide binding fold (known as the Walker A motif, defined by the G-X(4)-G-K-[TS] sequence), and its mutation is expected to yield both ATPase and endonuclease deficient ZRANB3. On the other hand, Asp1020 was identified in this study as an active site residue of the HNH endonuclease domain (Fig. 3c), and its mutation to tyrosine could be predicted to ablate the ZRANB3 endonuclease activity.

To investigate the functional significance of cancer associated *ZRANB3* mutations, we purified relevant ZRANB3 mutant proteins and tested their enzymatic activities. The majority of the tested cancer associated mutations yielded nucleolytically inactive ZRANB3 proteins (Fig. 8b,c). While in some mutants, such as R947Q, R947* and D1020Y, this was not related to the ATPase function (Fig. 8c, Table 2), in others the mutations caused ablation of both ATPase and endonuclease activities. Some mutations (K340T, F414C and C1041Hfs*13) resulted in insoluble proteins.

Collectively, these data suggest a high frequency of loss-of-function mutations in *ZRANB3* that may impact on its role in the replication stress response, and consequently on genomic stability.

## Discussion

Strategies to resolve replication blocks are critical for our understanding of basic molecular mechanisms that underlie the molecular basis of tumourigenesis. We have previously identified ZRANB3 as a structure-specific endonuclease that plays a role in the replication stress response^9^. A particularly interesting aspect of ZRANB3 function is that its nuclease activity strictly depends on active ATP hydrolysis (Figs 1e and 3c,d). Such dependence is unusual, but was previously observed in some bacterial nucleases (RecB and AddAB)^40^,^41^, and in eukaryotic DNA2 (ref. 42). Interestingly, the ATPase and nuclease active sites of ZRANB3 reside in two discrete domains within the ZRANB3 structure: the helicase core domain and the HNH domain, respectively.

Although many structure-specific nucleases have been studied in detail, ZRANB3 is relatively underexplored and the molecular basis of its nuclease activity lacked structural insight. Our detailed analyses highlighted similarities between ZRANB3 and two structure-specific endonucleases: T4 endonuclease VII (structurally related to ZRANB3 HNH domain) and FEN1 (structurally unrelated, but regulated by similar PCNA-dependent mechanisms). We further defined PCNA as a key regulator of ZRANB3 function, which recruits ZRANB3 to the sites of stalled DNA replication and stimulates its endonuclease activity. We also examined the way ZRANB3 interacts with PCNA by solving structures of PCNA:ZRANB3(PIP) and PCNA:ZRANB3(APIM) complexes. We found that, despite dissimilarities between the PIP box and the APIM motif consensus sequences, both motifs occupy the same binding site on the PCNA surface^30^ and share the basic topology with a characteristic 3_10_ helix. However, our data also point to the differences between the two motifs, and reveal specific interaction networks by which they form stable PCNA complexes.

Collectively, these results highlight the importance of specific molecular interactions in fine-tuning PCNA-binding affinities. This has important implications in the biological context, where PCNA coordinates a broad spectrum of activities involved in the genome replication and maintenance of its integrity^1^. To achieve this, PCNA must interact with a wide range of protein factors, either simultaneously or sequentially. PCNA therefore acts as a platform for the formation of specific molecular complexes, which are selected based on their relative PCNA-binding affinities^43^. The highest affinity reported to date is that of the p21 PIP box^5^, which enables it to displace replicative polymerases from the common binding site on the PCNA surface, and consequently to act as a cell cycle inhibitor. On the other hand, moderate PCNA-binding affinities seem to be well suited to support the formation of complexes of a more transient nature, such as those with translesion DNA polymerases or ZRANB3. Interestingly, increasing the PCNA-binding affinity of several PCNA partners in yeast resulted in severe *in vivo* phenotypic defects^44^, suggesting that the fragility of PCNA complexes plays an important role in conferring the appropriate biological activity.

Additional levels of complexity are achieved by the trimeric structure of the PCNA ring, and the fact that many PCNA binding proteins contain multiple PCNA binding motifs. The simultaneous occupation of PCNA protomers by separate PCNA binding motifs might therefore be a possibility for proteins like ZRANB3. This might not only be relevant for the stability of the complex, but also for its orientation and positioning on the DNA template. Finally, formation of specific protein:PCNA complexes is additionally modulated by post-translational modifications. Previous work demonstrated that polyubiquitinated PCNA recruits ZRANB3 to stalled replication forks, which is facilitated by the specific polyubiquitin binding motif NZF^9^,^10^, conserved among ZRANB3 proteins. Importantly, polyubiquitination of PCNA is known to regulate an error free pathway of cellular response to replication stress. This pathway, also known as template switching, uses the newly synthesized sister chromatid as a temporary template for DNA synthesis across the lesion^45^, and is conserved from yeast to humans^46^. The interactions through the NZF motif might therefore provide an additional level of regulation of the ZRANB3:PCNA complex and its activity at the replication fork.

Finally, our structural and biochemical findings allowed us to interpret the functional significance of cancer associated variants in *ZRANB3*. We analysed *ZRANB3* mutations associated with endometrial carcinomas and noted a high incidence of mutations predicted to yield functionally deficient proteins (Supplementary Table 2 and Fig. 8). Strikingly, the identified cancer-related mutations included the missense mutations targeting both of the ZRANB3 catalytic active sites: the ATPase active site contained within the helicase core, and the endonuclease active site in the HNH domain. Both of these mutations yielded nucleolytically inactive proteins (Fig. 8b,c), highlighting the biological significance of the ZRANB3 endonuclease function in this context.

Collectively, these results provide important insights into the structure and regulation of ZRANB3, and its association with PCNA. They also improve our understanding of the principal mechanisms employed by PCNA to interact with its protein partners. This seems particularly relevant, as emerging evidence suggests that targeting the PCNA binding surface might be a promising strategy in cancer therapeutics^47^,^48^. Detailed understanding of the dynamic nature of PCNA interactions might therefore open new possibilities for rational drug design.

## Methods

### Plasmids

Human ZRANB3 was cloned into a pFASTBac-His6-TEV vector for purification of the full-length ZRANB3 with an N-terminal His tag from insect cells. Point mutations and internal deletions in ZRANB3 gene were introduced using the QuickChange II site-directed mutagenesis kit (Stratagene). ZRANB3 HNH domain constructs (amino acids 948–1,067 and amino acids 871–1,079) were subcloned into pNH-TrxT vector for bacterial expression of proteins with N-terminal His and Trx tags (kindly provided by Opher Gileadi, SGC Oxford;^49^). PCNA was cloned into NcoI and XhoI sites of pET28a vector for the expression of the untagged protein, and into NdeI and XhoI sites for the expression of the protein with N-terminal His-tag. FEN1 was cloned into pET28a for the expression of the protein with C-terminal His-tag. Oligonulceotide sequences are listed in Supplementary Methods.

### Proteins

Full-length ZRANB3 and its mutants were expressed in High5 insect cells (Fisher Scientific). Expression of the ZRANB3 variants was initiated by infecting the cells (100 ml at 2 × 10^6^ cells per ml^−1^) with P1 virus at MOI (multiplicity of infection) ∼1. The cells were collected 48 h post infection and resuspended in lysis buffer (25 mM Tris-HCl at pH 8, 0.3 M NaCl, 10% glycerol, 1%Triton-X, protein inhibitor cocktail, 5 mM imidazole, 250 U benzonase ml^−1^ of lysate). The cleared lysate was passed through Ni-NTA beads, equilibrated with 50 mM Tris-Cl pH 8, 500 mM NaCl, 10 mM imidazole, 1 mM DTT. ZRANB3 was eluted with the same buffer containing 500 mM imidazole. To remove His-tag, the proteins were incubated with TEV protease overnight, reapplied to Ni-NTA beads, and then purified over a Superdex 200 10/300 column in 50 mM Tris-HCl pH 8, 500 mM NaCl and 1 mM DTT. Glycerol (final concentration 10%) was added to purified proteins before they were snap-frozen in liquid nitrogen and stored at −80 °C.

The ZRANB3 HNH domain (amino acids 948–1,067) used in protein crystallization was expressed from the pNH-TrxT vector by an overnight induction with 0.1 mM IPTG at 18 °C. Cells were lysed in lysis buffer (50 mM Tris-HCl pH 8, 500 mM NaCl, 10 mM imidazole, 4 mM β-mercaptoethanol, 2 mg ml^−1^ lysozyme, protease inhibitor cocktail (Roche) and 25 units ml^−1^ of benzonase (Sigma-Aldrich)) by applying three passages through a French-press at 15,000 Psi and insoluble material was pelleted for 90 min at 35,000 *g*. The cell extract was applied to Ni-NTA beads (Qiagen) equilibrated to lysis buffer, and the protein was eluted by increasing the concentration of imidazole to 500 mM. The protein was then incubated with TEV protease overnight, which removed His and Trx tags from the HNH domain. Cleaved protein was subsequently reapplied to Ni-NTA beads (to remove tags and TEV protease), and then purified over a Superdex S-200 (16/600) column in 20 mM Tris-HCl pH 7.5, 100 mM NaCl and 1 mM DTT. Homogenous HNH domain was concentrated to a final concentration of ∼30 mg ml^−1^ and used to set-up crystallization trials.

ZRANB3 HNH domain (amino acids 871–1,079) used for the electrophoretic mobility-shift assay was expressed from the pNH-TrxT vector, by a 4 h induction with 0.4 mM IPTG at 30 °C. The wild-type and mutant proteins were purified over Ni-NTA beads (Qiagen) analogously to the ZRANB3 HNH domain (amino acids 948–1,067). To remove imidazole, fractions containing the HNH domain were passed through a NAP-10 desalting column (GE healthcare), equilibrated with storage buffer (50 mM Tris-HCl pH 8.0, 500 mM NaCl, 1 mM DTT). Before freezing, glycerol was added (final concentration 10%), and the samples were snap-frozen in liquid nitrogen and kept in small aliquots at −80 °C.

Untagged PCNA for crystallization was expressed in *Escherichia coli* from pET28a vector during a 4 h induction with 0.4 mM IPTG at 30 °C. Cells were lysed in lysis buffer (50 mM Tris-HCl pH 8, 150 mM NaCl, 1 mM DTT, 2 mg ml^−1^ lysozyme, protease inhibitor cocktail (Roche) and 25 units ml^−1^ of benzonase (Sigma-Aldrich)) by applying tree passages through a French-press at 15,000 Psi and insoluble material was pelleted for 90 min at 35,000 *g*. The cleared supernatant was purified over a 10 ml Q-sepharose column equilibrated to Buffer A (50 mM Tris-HCl pH 8, 150 mM NaCl and 1 mM DDT). Proteins were eluted with 50 mM Tris-HCl pH 7.5, 1 mM DDT and a gradient of 150–550 mM NaCl. Fractions containing PCNA were pooled and loaded onto a 1 ml S-sepharose column equilibrated to Buffer A. The flow-through (containing PCNA) was passed through a 5 ml Hi-Trap Heparin column equilibrated with Buffer A. Finally, the flow-through (containing PCNA) was loaded onto a 5 ml Q-sepharose column equilibrated with Buffer A. PCNA was eluted in Buffer A with a gradient of 50–500 mM NaCl. Fractions containing PCNA were pooled, concentrated and loaded onto a Superdex S-200 (16/600) column equilibrated with storage buffer (20 mM Tris-HCl pH 8, 100 mM NaCl, 1 mM DTT). Homogenous PCNA was concentrated to a final concentration of ∼20 mg ml^−1^ and used to set-up crystallization trials.

His-tagged PCNA was expressed in *E. coli* from pET28a vector under the same conditions as the untagged PCNA. Cells were lysed in lysis buffer (50 mM Na-Phosphate pH 8, 500 mM NaCl, 1 mM β-mercaptoethanol, 10 mM imidazole, 2 mg ml^−1^ lysozyme, protease inhibitor cocktail (Roche) and 25 units ml^−1^ of benzonase (Sigma-Aldrich)).The protein was applied to Ni-NTA beads (Qiagen) equilibrated to lysis buffer, and eluted by increasing the concentration of imidazole to 500 mM. The protein was then applied to a 5 ml Q-sepharose column equilibrated to the PBS buffer containing 1 mM β-mercaptoethanol and eluted in the same buffer with 150–500 mM NaCl gradient. Fractions containing PCNA were purified over a Superdex S-200 (16/600) column equilibrated to the PBS buffer containing 1 mM β-mercaptoethanol. Before ITC measurements, PCNA was concentrated to ∼600 μM and extensively dialysed against the PBS buffer containing 1 mM β-mercaptoethanol, along with the ligand peptides.

### Crystallization and data collection and structure solution

Crystallization trials were performed with commercial screens using the sitting-drop vapour-diffusion method. Crystallization drops were set-up with the aid of a Mosquito Crystal robot (TTP Labtech) using 200 nl of protein solution plus 200 nl of reservoir solution in MRC two-well crystallization microplates (SWISSCI ‘MRC’) equilibrated against 75 μl of reservoir solution.

Crystals of the ZRANB3 HNH domain (amino acids 948–1,067) used for anomalous phasing grew in 20% PEG 6,000, 100 mM BICINE pH 9.0 at 4 °C and were soaked in crystallization solution with 5 mM K_2_PtCl_4_ for 10 min to obtain anomalous X-ray data for SAD experiments. Co-crystallization trials of HNH with dsDNA (5′-GCGATCGC-3′) produced crystals that grew in 20% PEG 10,000 and 100 mM HEPES pH 7.5 at 4 °C. Electron density for the dsDNA ligand could not be observed in the structure, even though crystals failed to form under identical crystallization conditions in the absence of the dsDNA ligand.

Peptides ^511^FTHFEKEKQHDIRSFFVPQPKK^532^ and ^515^EKEKQHDIRSFFVPQ^529^ were used for crystallization of PCNA:PIP complex. Co-crystals were obtained with both peptides, but peptide ^511^FTHFEKEKQHDIRSFFVPQPKK^532^ was more disordered at the C-terminus and residues beyond Phe526 were not visible. Co-crystals of PCNA and the PIP peptide (^515^EKEKQHDIRSFFVPQ^529^; 1:10 molar ratio) grew in 100 mM NaCl, 8% DMSO and 31% pentaerythritol propoxylate at 20 °C. Co-crystals of PCNA and the APIM peptide (^1066^SKHGSDITRFLVKK^1079^; 1:15 molar ratio) grew in 100 mM lithium sulfate, 30% polyvinylpyrrolidone and 100 mM HEPES pH 7.0 at 20 °C. All crystals were cryoprotected by a 2–5 s soak in crystallization solution with 20% glycerol, before being vitrified by submersion in liquid nitrogen.

X-ray data were collected at beamlines I03, I04 and I04-1 at the Diamond Light Source (Rutherford Appleton Laboratory, Harwell, UK) and data collection statistics for HNH, PCNA+PIP and PCNA+APIM are shown in Table 1.

Anomalous data of ZRANB3 HNH were collected at the Pt L-III absorption edge to 2.50 Å resolution. The structure was solved by SAD phasing, which located seven platinum ions and was followed by density modification and automated model-building. The resulting model was subsequently used to solve the 1.96 Å native data from a co-crystallization trial with dsDNA by molecular replacement. The PCNA+PIP and PCNA+APIM structures were solved by molecular replacement with a native human PCNA structure (PDB code: 1VYM) as the molecular replacement model.

X-ray data were processed using Xia2 (ref. 50). SAD phasing, initial density modification and automated model-building were carried out with AUTOSOL^51^. PHASER^52^ was used for phasing by molecular replacement. Subsequent density modification automated model building were implemented with PARROT^53^ and BUCCANEER^54^, respectively. Model building was carried out with COOT^55^ and real space refinement with REFMAC5 (ref. 56), coupled with automatically generated local non-crystallographic symmetry restraints and TLS refinement.

Figures were prepared with PyMOL (The PyMOL Molecular Graphics System, Version 1.8 Schrödinger, LLC) and UCSF Chimera^57^, using DSSP^58^ for secondary assignment.

### Nuclease assays

Oligonucleotides used in the nuclease assays (5′-AGGTCTCGACTAACTCTAGTCGTTGTTCCACCCGTCCAC-CCGACGCCACCTCCTG-3′ and Cy3 5′-end-labelled 5′-CGTCCTCCACCGCAGCCCACCTGCCCACCTAACTT-TAAATCCGACCGTGCCAGC-3′) were incubated in 10 mM Tris-HCl pH 7.5, 50 mM NaCl for 3 min at 94 °C, after which the reactions were allowed to gradually cool down.

The nuclease assays with ZRANB3 was performed essentially as described in ref. 9. Briefly, reaction mixtures containing the indicated nuclease (200 nM) and fluorescently labelled DNA substrate (10 nM) in 10 μl of reaction buffer (50 mM Tris-HCl; 50 mM NaCl; 5 mM MgCl_2_; 2 mM ATP (Sigma); pH 7.5) were incubated at 30 °C for 60 min. The reactions were stopped by incubation with 0.1% SDS and 4 mg ml^−1^ of proteinase K at 30 °C for 45 min. The reactions were then mixed with sucrose (10% final concentration) and resolved in a 10% polyacrylamide gel in 0.5 × TBE buffer (45 mM Tris-borate, 1 mM EDTA; pH 7.5). The fluorescent DNA species were visualized and quantified using Pharos Mx imager and Quantity one software (BioRad).

### ATPase assays

The ATPase activity of ZRANB3 was examined by two methods: one, based on resolution of radioactive ^32^Pi product by thin layer chromatography (TLC), and the other, measuring time-dependent consumption of ATP using an NADH-coupled assay.

The TLC-based ATPase assay was performed essentially as described^9^. Briefly, the reactions were performed in presence of 10 μM ATP, 0.1 μCi of [γ-^32^P]ATP (3,000 Ci m mol^−1^) (Perkin Elmer), 50 mM Tris-HCl pH 7.5, 5 mM MgCl_2_, 200 nM flap DNA and 162 nM of ZRANB3 proteins in a 5-μl reaction volume. Where indicated, DNA was added to the reaction. After a 30 min incubation at 30 °C, the reactions were stopped by the addition of 50 mM EDTA. In a set of control experiments, 5.27 μM RecA recombinase (in the presence or absence of virion ΦX-174 DNA; 115 nM) was used according to the manufacturer’s instructions. Reaction products were spotted onto a polyethyleneimine (PEI)-cellulose plate (Macherey-Nagel, Polygram CEL 300 PEI UV254^−1^) and developed in 0.15 M LiCl and 0.15 M formic acid. Dried plates were exposed onto an X-ray film.

The NADH-coupled ATPase assay is based on a reaction in which the regeneration of hydrolysed ATP is coupled to the oxidation of NADH^59^. It was used as described^59^, with minor modifications. ZRANB3 (150 nM) was added to a 150 μl reaction containing 25 mM Tris-Cl pH 7.5, 13 mM MgCl_2_, 1.8 mM DTT, 100 μg ml^−1^ BSA, 3 mM phosphoenolpyruvate, 36 U ml^−1^ pyruvate kinase, 20 U ml^−1^ lactate dehydrogenase, 0.5 μM flap DNA, and 5 mM ATP, with 750 μM NADH added immediately before ZRANB3. Reactions were mixed for 1 min on a shaker at 23 °C and analysed by measuring A_340_ _nm_ at 20 s intervals during 60 min, on a Spectramax 250 microplate spectrophotometer at 24 °C. Linear titration of freshly prepared NADH was performed to obtain a standard curve for units of absorbance mM^−1^ NADH. ATPase rates were calculated from data collected between time points where product formation was linear with respect to time.

### Isothermal titration calorimetry

PCNA and peptides used in our study were extensively dialysed against PBS containing 1 mM β-mercaptoethanol. The concentration of the peptides was determined at A205 nm, using a molar absorptivity calculated specifically from the amino-acid sequence^60^.

The measurements were performed at 25 °C using MicroCal PEAQ-ITC (Malvern). In a standard experiment, the reaction chamber was filled with a 15 μM solution of PCNA. The peptides were injected stepwise (20 injections of 2 μl). Concentrations of the peptides in the syringe were as follows: p21 (147 μM); Polι (398 μM); FEN1 (410 μM); ZRANB3 PIP (311 μM); ZRANB3 APIM (327 μM). The obtained results were processed and analysed using MicroCal PEAQ-ITC Analysis Software. The free Gibbs energy (Δ*G*), Enthalpy change (Δ*H*) and stoichiometry (*N*) were determined by Levenberg–Marquardt curve-fitting method employing single set of independent binding sites model. Association constant (*K*_A_) was determined using the following equation Δ*G*=−RTln (*K*_A_). Association constant (*K*_D_) is inverse function of the dissociation constant *K*_*A*_. Finally, the entropy difference (−TΔS) was obtained from the following equation: Δ*G*=Δ*H*–TΔS.

### PCNA pull-downs

Biotinylated PIP-box and APIM-motif peptides were synthesized by Genscript. Magnetic streptavidin beads (Life Technologies) were used to immobilize the peptides, which were then washed with 50 mM Tris-HCl pH 8, 150 mM NaCl, 1 mM DTT and 0.2% Triton-X. The beads were then incubated with His-tagged recombinant PCNA for 1 h at 4 °C. The beads were subsequently washed with the same buffer to remove unbound PCNA, after which they were boiled in the SDS-PAGE loading buffer, and analysed by anti-PCNA Western blotting (ab18197, 1:1,000 dilution).

### Electrophoretic mobility-shift assay

DNA substrate was prepared by mixing a 1.5-fold excess of unlabelled oligonucleotide (5′-CGACTTCCGGTAGCACGTAGCAGCGGCTCGCCACGAACTGCACTCTAGGC-3′) over the 5′ ^32^P-labelled oligonucleotide (5′-CCTCGATCCTACCAACCAGATGACGCGCTGCTACGTGCTACCGGAAGTCG-3′). The oligonucleotides were annealed in 10 mM Tris-HCl pH 7.5, 50 mM NaCl by slow cooling of reactions from 94 °C. Purified Trx-tagged wild-type and mutant HNH domains (amino acids 871–1,079) were incubated with radioactively labelled substrate (1 nM) at 4 °C in 15 μl of reaction buffer (50 mM Tris-HCl pH 7.5, 5% glycerol, 4 mM EDTA, 1 mM DTT and 0.1 mg ml^−1^ BSA) for 30 min. After the incubation, the reaction mixtures were resolved in a 5% native polyacrylamide gels in 0.5 × TBE buffer (45 mM Tris-borate, 1 mM EDTA; pH 7.5) at 4 °C. Resolved gels were dried and visualized by autoradiography.

### Immunofluorescence

U2OS cells (ATCC) were seeded onto glass coverslips and transfected with the YFP constructs using Lipofectamine 2000 (Life Technologies). The following day, the cells were first washed with PBS, and then incubated in pre-extraction buffer (10 mM PIPES, 300 mM sucrose, 3 mM MgCl_2_, 20 mM NaCl, 0.5% Triton-X) for 5 min at −20 °C. They were then fixed in 4% paraformaldehyde for 15 min, permeabilized with 0.5% Triton-X for 5 min, washed in PBS and further incubated in 2% BSA for 30 min. The cells were then stained with the rabbit PCNA antibody (Abcam, ab18197, 1:300 dilution), washed in PBS, and incubated with the anti-rabbit secondary antibody coupled to Alexa Fluor 594 (Life Technologies). The coverslips were mounted in ProLong Gold anti-fade reagent with DAPI (Life Technologies) and analysed by confocal microscopy.

For studies involving YFP-NLS-PIP and YFP-NLS-APIM constructs, the following sequences were introduced to the C-terminus of YFP: GFMRAPKKKRKVGGFTHFEKEKQHDIRSFFVPQPKK (for YFP-NLS-PIP), GFMRAPKKKRKVGGFTHFEKEKAHDIRSAAVPQPKK (for YFP-NLS-PIP*), GFMRAPKKKRKVGGQVRRQSLASKHGSDITRFLVKK (for YFP-NLS-APIM) and GFMRAPKKKRKVGGQVRRQSLASKHGSDITRALVKK (for YFP-NLS-APIM*).

For the recruitment of ZRANB3 to stalled replication forks, U2OS cells were seeded onto glass coverslips and transfected with YFP-ZRANB3 constructs. The following day they were treated with UV (40 J m^−2^), and allowed to recover for 6 h. This was followed by pre-extraction and staining with the PCNA antibody, exactly as described above. Finally, the samples were analysed by confocal microscopy. For each experiment, more than 100 cells were counted, and the results from three independent transfections were pooled for statistical analyses.

### Live-cell imaging by laser microirradiation

U2OS cells were grown in glass-bottomed dishes and transfected with the appropriate YFP constructs using Lipofectamine 2000 (Life Technologies). They were sensitized with 10 μM BrdU for 16 h at 37 °C. Laser microirradiation was carried out on an Olympus confocal microscope equipped with an environmental chamber. Cells showing moderate levels of expression were systematically chosen using identical 488-nm laser settings. DNA damage was induced by 405 nm laser and recruitment of the proteins was monitored by live cell imaging.

### Bioinformatic analyses of pathogenicity

Pathogenicity of *ZRANB3* variants was evaluated by PolyPhen-2 (ref. 61), SIFT^62^, Mutation Assessor^63^ and Mutation Taster2 (ref. 64).

### Data availability

Coordinates and structure factors were deposited in the Protein Data Bank with accession codes: 5MKW (Crystal structure of the human ZRANB3 HNH domain), 5MLO (Crystal structure of human PCNA in complex with the ZRANB3 PIP box peptide) and 5MLW (Crystal structure of human PCNA in complex with the ZRANB3 APIM motif peptide). All other data are available from the corresponding author on reasonable request.

## Additional information

**How to cite this article:** Sebesta, M. *et al*. Structural insights into the function of ZRANB3 in replication stress response. *Nat. Commun.* **8**, 15847 doi: 10.1038/ncomms15847 (2017).

**Publisher’s note:** Springer Nature remains neutral with regard to jurisdictional claims in published maps and institutional affiliations.

## Supplementary Material

Supplementary Information Supplementary figures, supplementary tables and supplementary methods.

## Figures and Tables

**Table 1 t1:** Summary of crystallographic statistics.

	**HNH-Pt**	**HNH**	**PCNA:PIP**	**PCNA:APIM**
*Data collection*				
Wavelength (Å)/beam line	1.0721/I03	0.9174/I04-1	1.1402/I04	1.2822/I03
Detector	Pilatus3 6M	Pilatus 2M	Pilatus 6M-F	Pilatus3 6M
Space group	*P*2_1_ 2_1_ 2_1_	*P*2_1_ 2_1_ 2_1_	*P*2_1_ 2_1_ 2_1_	*P*3_1_ 2_1_
*a* (Å)	54.14	56.84	68.14	84.63
*b* (Å)	61.14	67.55	86.80	84.63
*c* (Å)	75.34	92.16	156.69	201.80
*α* (°)	90.00	90.00	90.00	90.00
*β* (°)	90.00	90.00	90.00	90.00
*γ* (°)	90.00	90.00	90.00	120.00
Content of asymmetric unit	2	2	3	3
Resolution (Å)	27.59–2.39 (2.45–2.39)	54.48–2.00 (2.05–2.00)	78.34–1.96 (2.01–1.96)	73.29–2.45 (2.55–2.45)
*R*_sym_ (%)*	6.6 (65.0)	20.8 (186.2)	8.4 (176.3)	10.4 (129.7)
*I*/*σ* (*I*)	15.4 (2.9)	11.0 (1.4)	20.2 (1.5)	15.1 (2.0)
Completeness (%)	99.7 (99.3)	99.9 (99.7)	100.0 (100.0)	100.0 (100.0)
Redundancy	6.4 (6.6)	13.2 (11.9)	13.0 (13.1)	11.2 (9.7)
CC_1/2_ (%)	(81.5)	(52.2)	(56.6)	(56.5)
Number of unique reflections	10,367 (748)	24,666 (1,783)	67,566 (4,503)	31,686 (3,528)
				
*Refinement*				
*R*_cryst_ (%)†	25.7	19.2	21.1	22.1
*R*_free_ (%)‡	30.5	23.6	25.9	26.4
Rmsd bond length (Å)	0.017	0.013	0.010	0.011
Rmsd bond angle (°)	1.87	1.50	1.40	1.42
				
*Number of atoms/ions*				
Protein	1,253	1,991	6,025	5,962
Peptide	0	0	286	255
Water	30	232	390	96
Platinum	7	0	0	0
Zinc	0	2	0	0
Sodium	0	0	4	0
				
*Average B factor (Å*^*2*^)				
Protein	64.3	30.3	42.2	67.3
Peptide	NA	NA	60.7	76.2
Water	59.5	38.3	46.6	56.2
Platinum	216.9	NA	NA	NA
Zinc	NA	23.7	NA	NA
Sodium	NA	NA	56.6	NA
				
*Ramachandran plot*				
Favoured	93.6	96.8	97.2	95.7
Allowed	5.7	3.2	2.7	3.9
Disallowed	0.7	0	0.1	0.4

APIM, AlkB homologue 2 PCNA Interacting Motif; HNH, His-Asn-His; NA, not applicable; PCNA, Proliferating Cell Nuclear Antigen; PIP, PCNA-Interacting Protein; Rmsd, root mean square deviation.

a Data for the highest resolution shell are given in parentheses.

^*^R_sym_=Σ|/-</>|/Σ/, where / is measured density for reflections with indices *hkl*.

^†^*R*_cryst_=Σ||Fobs|–|Fcalc||/Σ|Fobs|.

^‡^*R*_free_ has the same formula as *R*_cryst_, except that calculation was made with the structure factors from the test set.

**Table 2:** Average rates of ATP hydrolysis determined by an NADH-coupled assay according to ref. 59.

**Enzyme**	**ATP hydrolysis rate (μmol** **l**^−**1**^ **min**^−**1**^**)**	***k* (min**^−**1**^**)**
WT ZRANB3	16.59±0.34	110.65±2.27
		
*ZRANB3-specific HNH insert deletion mutants*		
ZRANB3-Δ975–1013	5.76±0.03	38.41±0.23
ZRANB3-Δ972–1010	4.83±0.90	32.24±6.00
		
*Basic residues HNH mutants*		
K984A	20.93±2.01	139.56±13.45
R987A	6.34±0.40	42.31±2.70
K988A	18.83±1.94	125.55±12.95
K998A	15.12±0.48	100.85±3.22
R1009A	7.49±2.57	49.94±17.14
K1046A, R1048A	19.38±1.74	129.23±11.62
		
*HNH active site mutants*		
H1015A	18.67±2.24	124.46±14.96
D1020A	12.94±0.67	86.32±4.49
H1021A	18.59±1.98	123.95±13.19
N1036A	14.05±2.46	93.71±16.43
H1045A	8.91±0.66	59.42±4.40
		
*PCNA binding mutants*		
PIP*	20.71±0.86	138.10±5.76
ΔAPIM	20.24±0.70	134.95±5.22
PIP* ΔAPIM	20.40±4.40	136.02±29.39
		
*Cancer associated mutants*		
T66A	0.45±0.03	3.02±0.21
R169H	ND	ND
G401D	ND	ND
R947Q	10.24±1.15	68.26±7.70
R947*	9.50±0.64	63.36±4.31
D1020Y	11.60±3.30	77.36±22.04

APIM, AlkB homologue 2 PCNA Interacting Motif; HNH, His-Asn-His; ND, not defined; PCNA, Proliferating Cell Nuclear Antigen; PIP, PCNA-Interacting Protein.

ATP hydrolysis rate was calculated using the molar absorption coefficient for NADH in water (6220 L mol^−1^ cm^−1^) and the light path of 0.424 cm.
