# Supplementary material for: Structural insights into the function of ZRANB3 in replication stress response
Source: Nat Commun. 2017 Jun 16;8:15847. doi: 10.1038/ncomms15847 (PMC5481773; doi:10.1038/ncomms15847)
Supplement: Supplementary Information — Supplementary figures, supplementary tables and supplementary methods. [file ncomms15847-s1.pdf]

Type of file: PDF
Size of file: 0 KB
Title of file for HTML: Supplementary Information
Description: Supplementary figures, supplementary tables and supplementary methods.

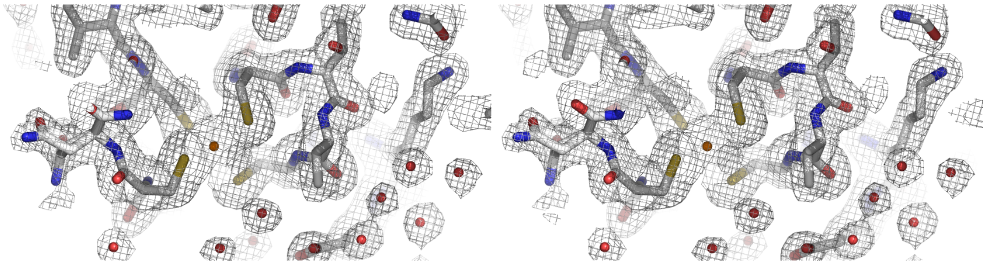

Supplementary Figure 1. The HNH domain electron density map
Stereo view of the HNH domain electron density map. Shown is a non-catalytic zinc-finger coordinated with four cysteine residues. 2Fo−Fc density map is contoured at 1.0 σ and colored grey.

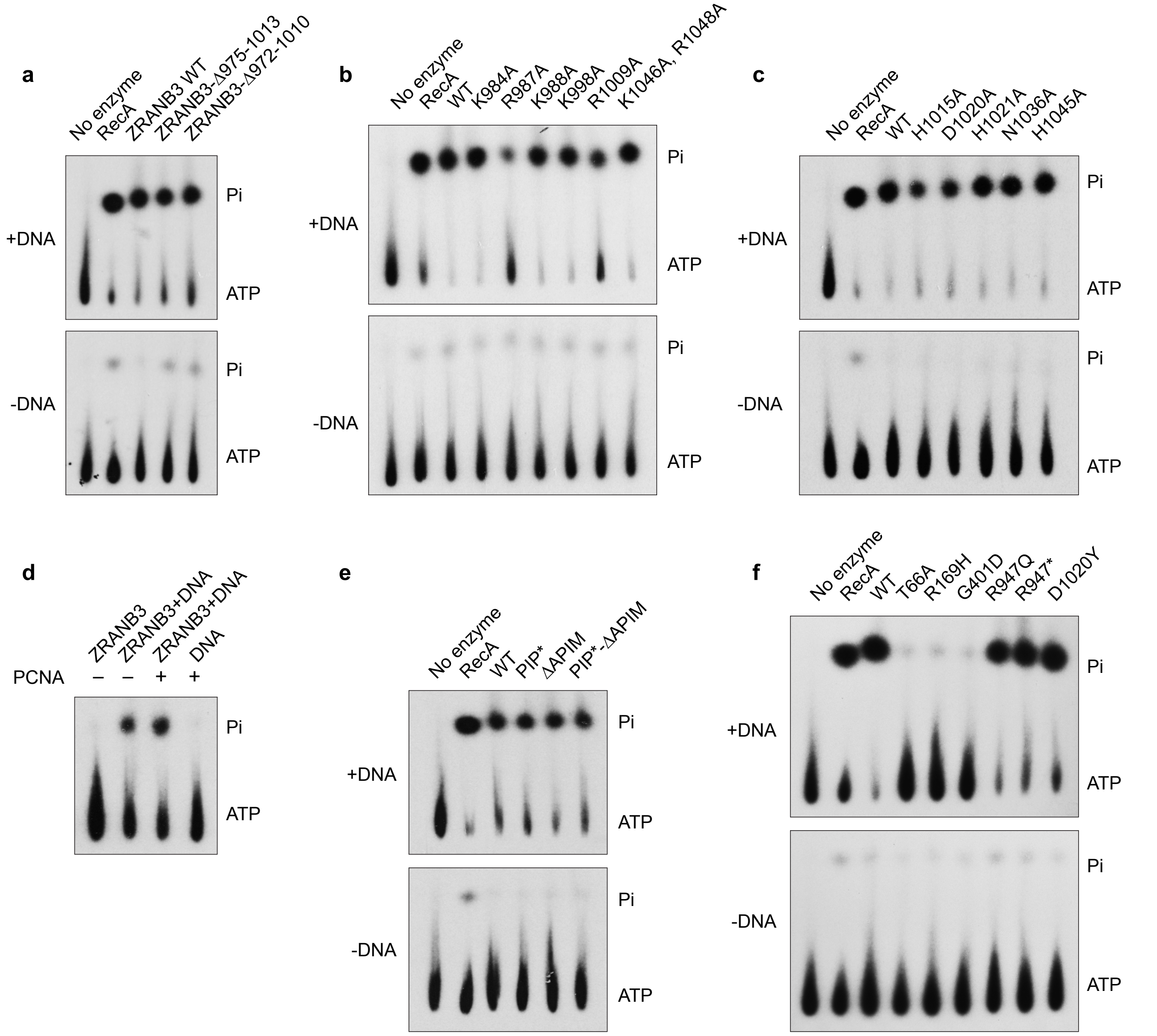

Supplementary Figure 2. ATPase activities of ZRANB3 proteins

a.- f. ATPase assay with the wild-type and mutant ZRANB3 proteins. RecA was used as a control. Recombinant proteins were incubated with ^{32}P-labeled ATP in the absence or presence of DNA (splayed DNA duplex). Reaction products were resolved by thin-layer chromatography. Chromatographic mobilities of the ATP substrate and Pi product are indicated. **a.** ATPase assay with the two mutants containing deletions of the ZRANB3-specific helical domain (ZRANB3-Δ975-1013 and ZRANB3-Δ972-1010). **b.** ATPase assay with the basic residues HNH mutants. **c.** ATPase assay with the HNH active site mutants. **d.** PCNA does not stimulate ATPase activity of ZRANB3. **e.** ATPase assay with the PCNA binding mutants. **f.** ATPase assay with the cancer associated ZRANB3 mutants.

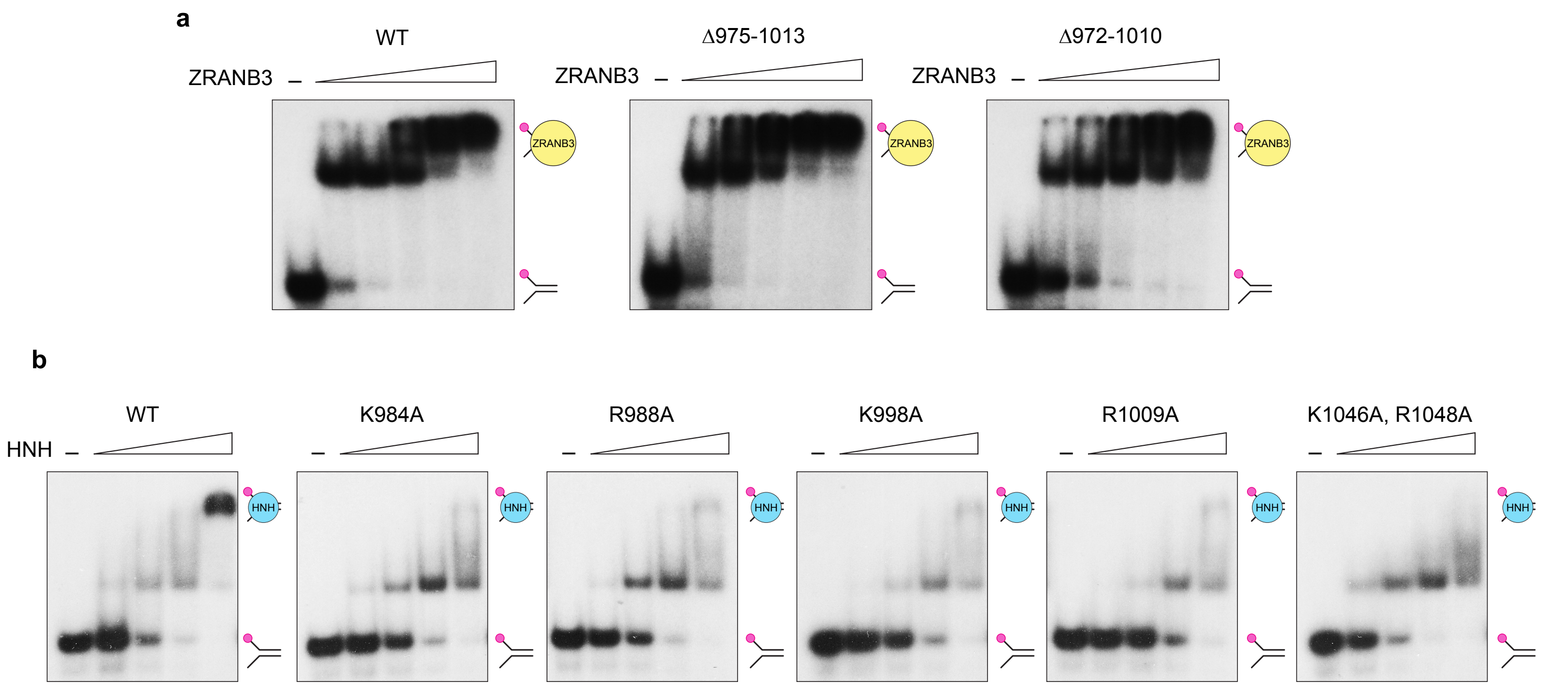

Supplementary Figure 3. Electrophoretic mobility-shift assays

a. Electrophoretic mobility-shift assay with wild type and mutant ZRANB3 proteins. Increasing concentrations (11, 22, 44, 88, 176 nM) of purified wild type and mutant ZRANB3 proteins were incubated with radioactively labelled substrate DNA and resolved by polyacrylamide gel electrophoresis.

b. Electrophoretic mobility-shift assay with the HNH domain wild type and mutant proteins. Increasing concentrations (4, 8, 16, 32 μM) of purified Trx-tagged wild type and mutant HNH domains (amino acids 871-1079) were incubated with radioactively labelled substrate DNA and resolved by polyacrylamide gel electrophoresis.

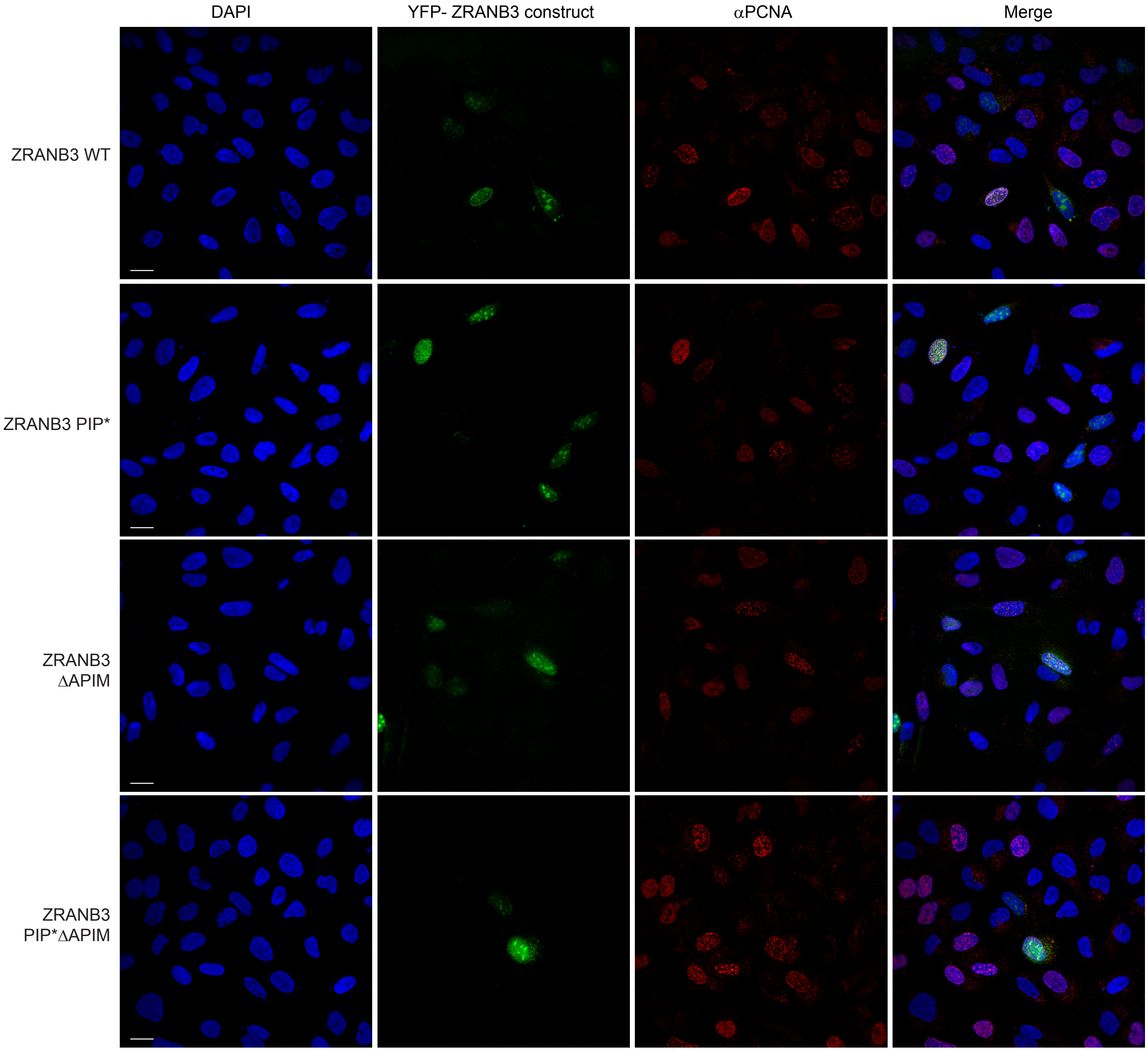

Supplementary Figure 4. Colocalization of ZRANB3 with PCNA
Accumulation of ZRANB3 at sites of ongoing DNA replication in the absence of exogenous DNA damage. U2OS cells were transfected with YFP-ZRANB3 constructs, treated with a pre-extraction buffer (see Materials and methods), fixed and stained with PCNA antibody. The percentage of cells containing ZRANB3 foci that colocalize with PCNA was determined and shown in Fig. 5e. Scale bar: 20 μm.

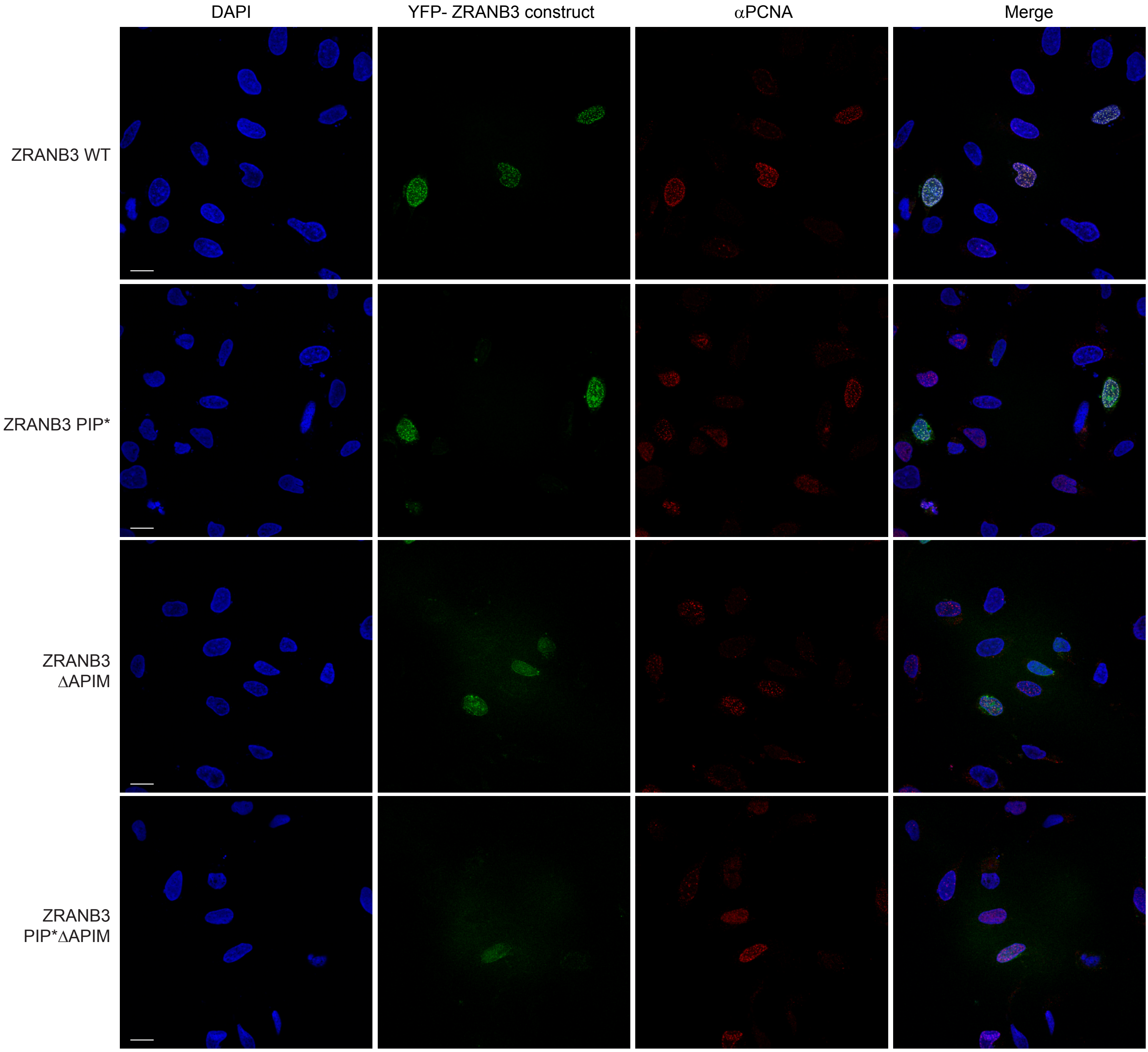

Supplementary Figure 5. Colocalization of ZRANB3 with PCNA after UV damage
Accumulation of ZRANB3 at sites of stalled DNA replication following DNA damage. U2OS cells were transfected with YFP-ZRANB3 and exposed to UV irradiation. After 6 h, cells were treated with a pre-extraction buffer (see Materials and methods), fixed and stained with PCNA antibody. The percentage of cells containing ZRANB3 foci that colocalize with PCNA was determined and shown in Fig. 5e. Scale bar: 20 μm.

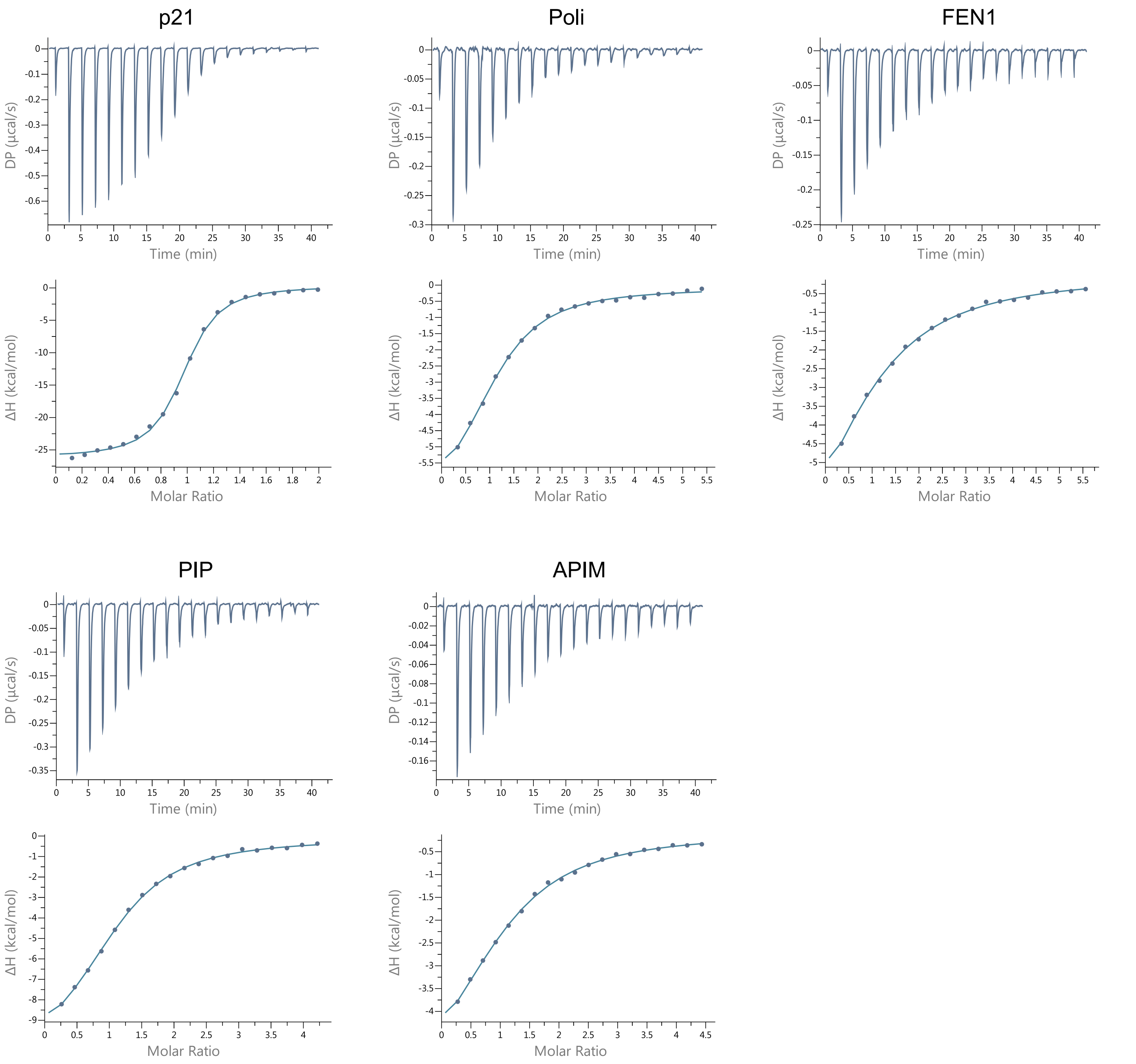

Supplementary Figure 6. Isothermal titration calorimetry measurements
Isothermal titration calorimetry measurements of PCNA with the indicated peptides. Shown are thermograms and the binding isotherms from the integrated thermogram fits, with the one-site model (as analysed by MicroCal PEAQ-ITC Analysis Software).

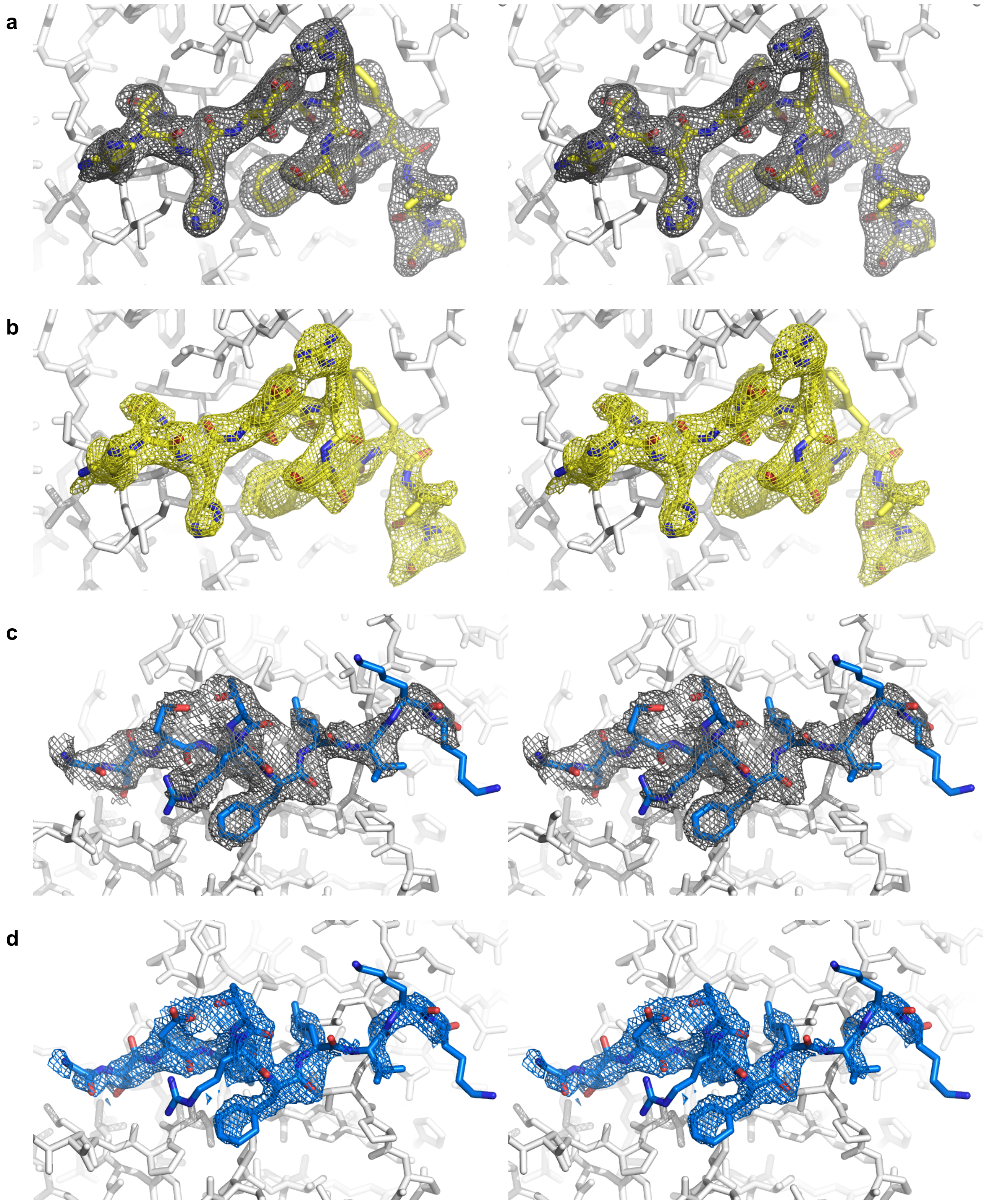

Supplementary Figure 7. Electron density maps of PIP box and APIM motif peptides
a. Stereo view of the PIP box peptide electron density map (grey). 2Fo−Fc density map is contoured at 1.0 σ and colored grey. PIP box peptide is shown as yellow and PCNA as white sticks. **b**. Unbiased electron density map for the PIP peptide (yellow), calculated after molecular replacement using a trimeric PCNA structure (PDB code 1VYM) as a search model and 50 cycles of initial refinement with jelly-body refinement in REFMAC5. PIP box peptide is shown as yellow and PCNA as white sticks. **c**. Stereo view of the APIM motif peptide electron density map (grey). 2Fo−Fc density map is contoured at 1.0 σ and colored grey. APIM motif peptide is shown as blue and PCNA as white sticks. **d**. Unbiased electron density map for the APIM peptide (blue), calculated after molecular replacement using a trimeric PCNA structure (PDB code 1VYM) as a search model and 50 cycles of initial refinement with jelly-body refinement in REFMAC5. APIM motif peptide is shown as yellow and PCNA as white sticks.

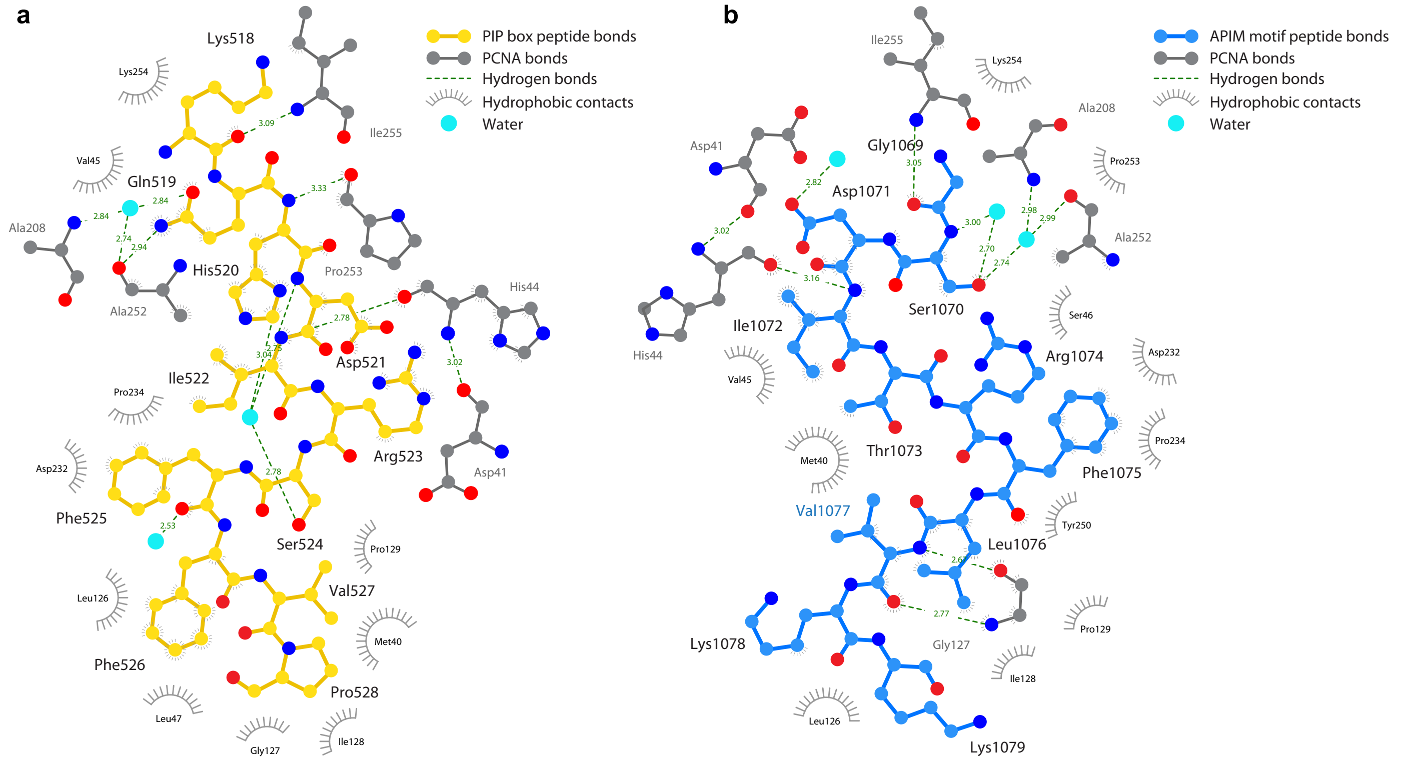

c

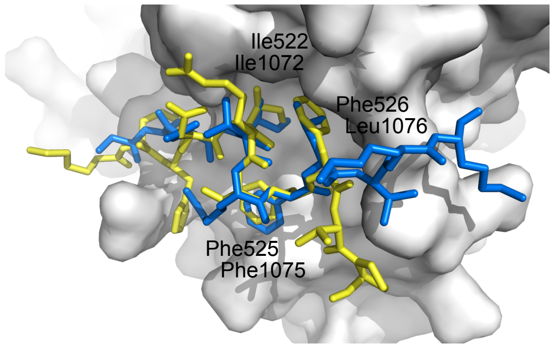

Supplementary Figure 8. Interactions of ZRANB3 PCNA binding peptides with PCNA

a. Schematic diagram of PCNA-PIP box peptide interactions generated by LIGPLOT.

b. Schematic diagram of PCNA-APIM motif peptide interactions generated by LIGPLOT.

c. Superimposition of the PIP box (yellow sticks, residues 518-528) and APIM motif (blue sticks, residues 1069-1079) peptides bound to PCNA (grey sufrace.)

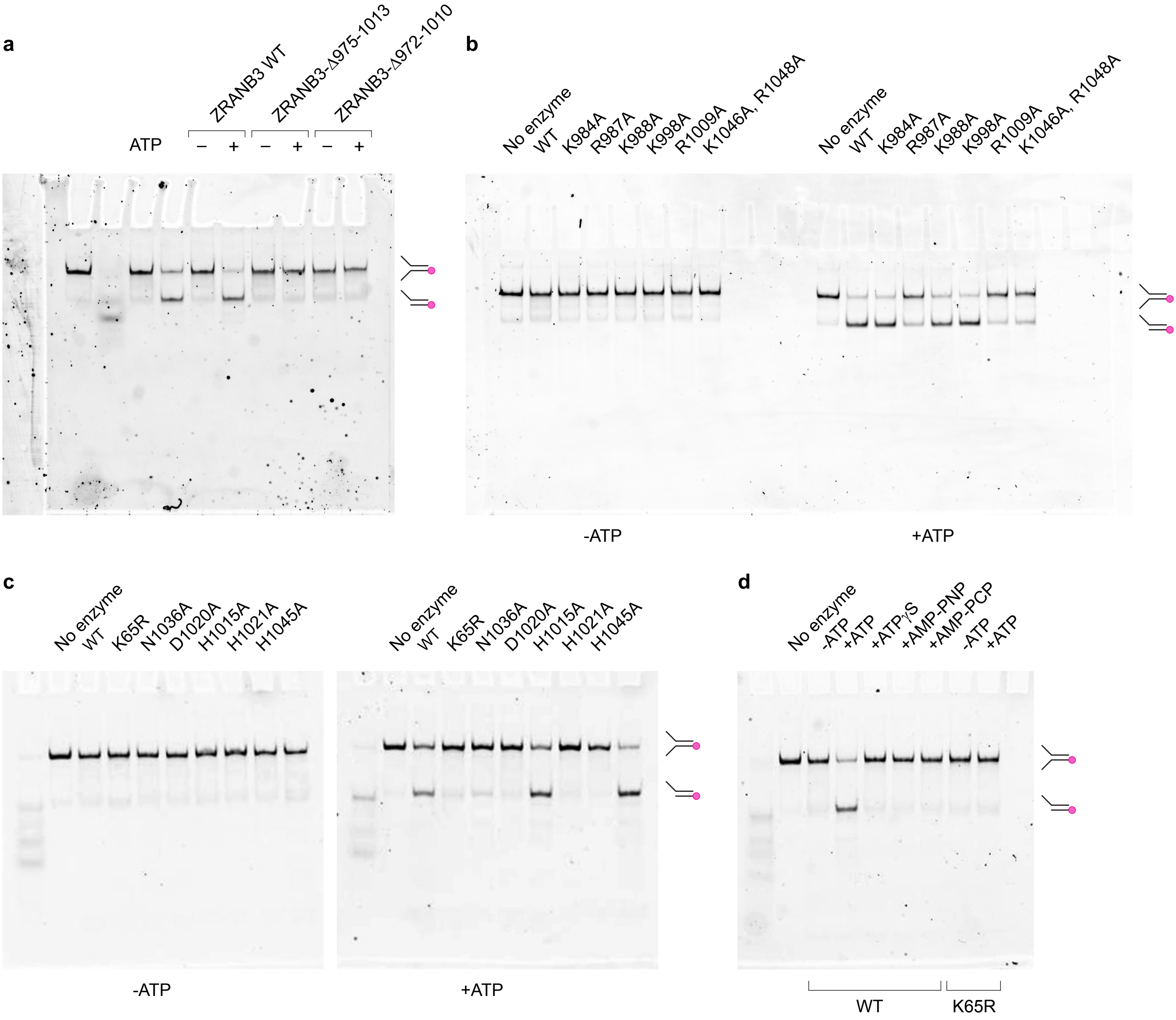

Supplementary Figure 9. Uncropped scans of gels shown in Figs. 1-3.

a. Uncropped scan of Fig. 1e. **b**. Uncropped scan of Fig. 2d. **c**. Uncropped scan of Fig. 3c. **d**. Uncropped scan of Fig. 3d.

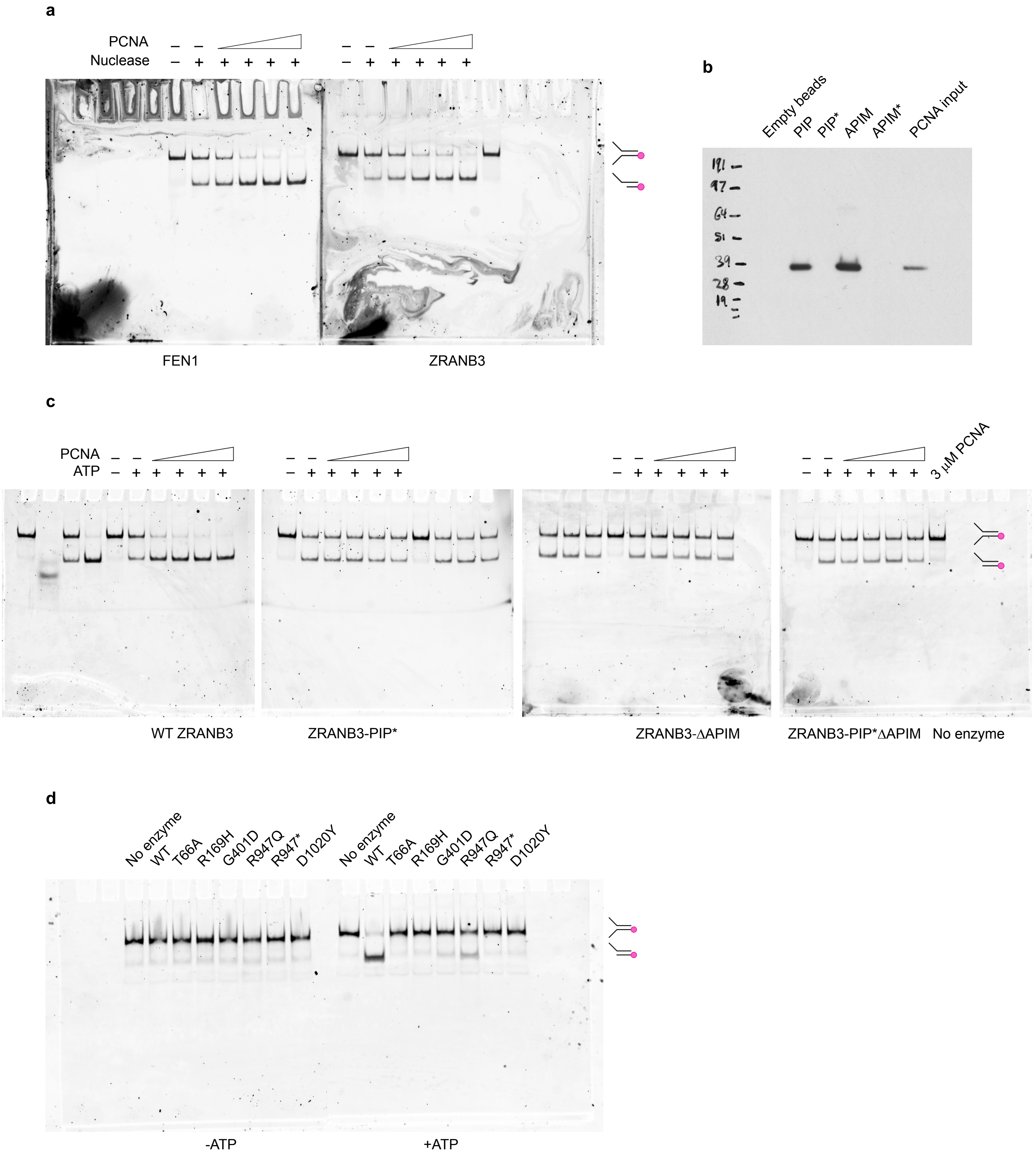

Supplementary Figure 10. Uncropped scans of gels shown in Figs. 4-8.

a. Uncropped scan of Fig. 4a. **b**. Uncropped scan of Fig. 4c. **c**. Uncropped scan of Fig. 4d. **d**. Uncropped scan of Fig. 8b.

a

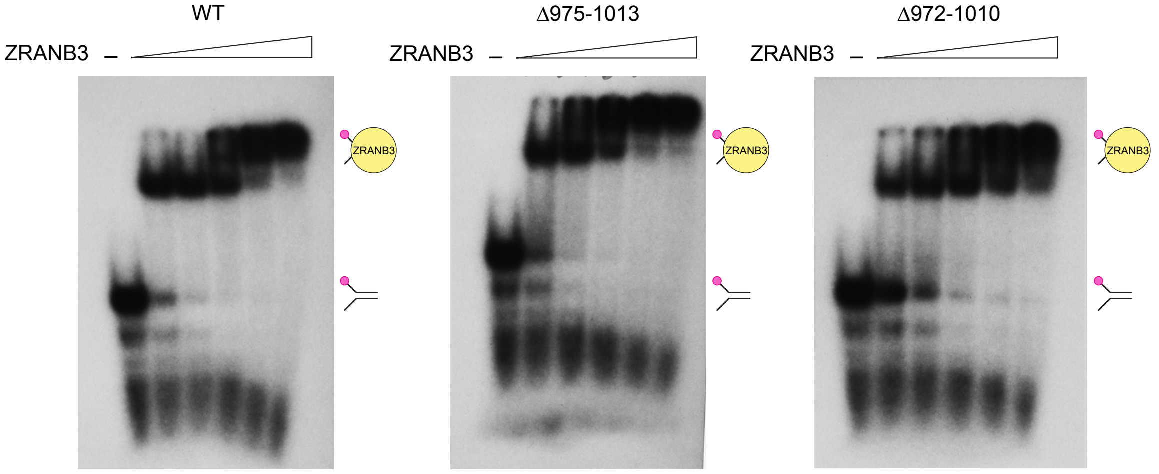

b

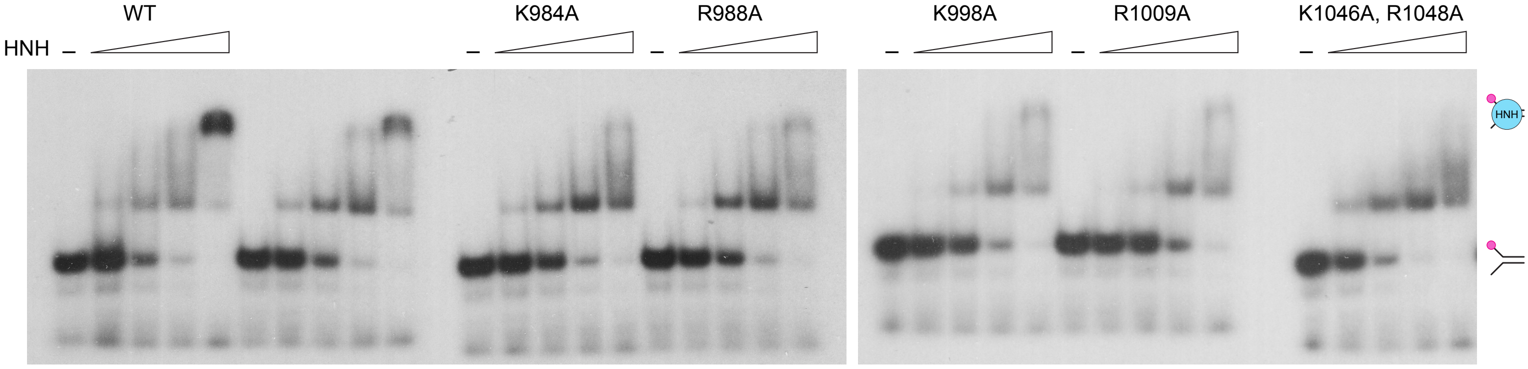

Supplementary Figure 11. Uncropped scans of gels shown in Supplementary Fig. 3.
a. Uncropped scan of Supplementary Fig. 3a. **b**. Uncropped scan of Supplementary Fig. 3b.

Supplementary Table 1. Isothermal titration calorimetry measurements of PCNA with the indicated peptides.

Peptide	N (stoichiometry)	K_D (μM)	ΔG (kcal mol-1)	ΔH (kcal mol-1)	-TΔS (kcal mol-1)
p21	0.944 ± 0.006	0.275 ± 0.0248	-89.5	-26.4 ± 0.343	17.5
Poli	1.08 ± 0,027	5.45 ± 0.571	-7.18	-7.11 ± 0.338	-0.062
FEN1	1.02 ± 0.095	17.3 ± 2.93	-6.5	-10.5 ± 1.68	-39.9
ZRANB3 PIP	1.08 ± 0.016	4.8 ± 0.352	-7.26	-11.1 ± 0.325	3.88
ZRANB3 APIM	1.04 ± 0.036	9.24 ±1.14	-6.87	-6.34 ± 0.475	-0.532

Peptide sequences: p21 ^{135}DSQGRKRRQTSMTDFYHSKRRL^{157}, Polι ^{438}LKALNTAKKGLIDYYLMPSLST^{459}, FEN1 ^{329}SKSRQGSTQGRLDDFFKVTGSL^{350}, ZRANB3 (PIP) ^{511}FTHFEKEKQHDIRSFFVPQPKK^{532}, ZRANB3 (APIM) ^{1058}QVRRQSLASKHGSDITRFLVKK^{1079}.

Supplementary Table 2. Bioinformatic analysis of *ZRANB3* variants by pathogenicity prediction programmes.

**Algorithm**	PolyPhen-2		SIFT		Mutation Assessor		Mutation Taster	
**Mutation**	Score	Prediction	Score	Prediction	FI Score	Impact	Score	Prediction
**T66A**	1.000	Probably damaging	0	Damaging	4.54	High	58	Disease causing
**R169H**	1.000	Probably damaging	0	Damaging	3.47	Medium	29	Disease causing
**R313C**	0.124	Benign	0	Damaging	2.565	Medium	180	Disease causing
**K340T**	1.000	Probably damaging	0	Damaging	4.29	High	78	Disease causing
**G401D**	1.000	Probably damaging	0.01	Damaging	4.49	High	94	Disease causing
**F414C**	1.000	Probably damaging	0	Damaging	3.185	Medium	205	Disease causing
**K706T**	0.002	Benign	0.1	Tolerated	1.095	Low	78	Polymorphism
**R947Q**	1.000	Probably damaging	0.01	Damaging	2.48	Medium	43	Disease causing
**S997P**	0.987	Probably damaging	0	Damaging	2.7	Medium	74	Polymorphism
**D1020Y**	1.000	Probably damaging	0	Damaging	4.425	High	160	Disease causing

The PolyPhen-2 score ranges from 0.0 (tolerated) to 1.0 (deleterious). The software uses structural and comparative evolutionary considerations to predict the possible impact of amino acid substitutions [61].

SIFT score ranges from 0 to 1. The amino acid substitution is predicted damaging is the score is ≤ 0.05, and tolerated if the score is > 0.05. SIFT prediction is based on the degree of conservation of amino acid residues in sequence alignments derived from closely related sequences, collected through PSI-BLAST [62].

The functional impact (FI) score in mutation assessor is derived from multiple sequence alignments of sequence homologs. Larger scores indicate more likely functional impact of a mutation [63].

The Mutation Taster score is taken from the Grantham Matrix for amino acid substitutions and reflects the physicochemical difference between the original and the mutated amino acid. It ranges from 0.0 to 215. The software uses the frequency of the respective amino acid exchange in known disease causing mutations and polymorphisms for the classification. It predicts an alteration as one of four possible types: disease causing - i.e. probably deleterious; disease causing automatic - i.e. known to be deleterious; polymorphism - i.e. probably harmless; and polymorphism automatic - i.e. known to be harmless [64].

Supplementary Table 3. Selection of *ZRANB3* mutations associated with endometrial carcinomas.

Mutation	Location	Conservation	Impact
T66A	Helicase core, Walker A motif	Yes	ATPase and endonuclease deficient ZRANB3
E97*	Helicase core	No	Functional null
K340T	Helicase core	Yes	Unknown
G401D	Helicase core	Yes	Unknown
F414C	Helicase core	Yes	Unknown
R947*		No	Truncated ZRANB3, endonuclease deficient
D1020Y	HNH domain, active site	Yes	Endonuclease deficient
C1041Hfs*13	HNH domain, zinc-finger	Yes	Truncated ZRANB3, endonuclease deficient

C1041Hfs*13 denotes a frameshift mutation which changes Cys1041 to His, and introduces a stop codon at position 1053.

Supplementary Methods

Oligonucleotides used in this study:

ZRANB3 HNH domain into pNH-TrxT
TACTTCCAATCCATGTCTAATAACAGTTACCTG and GCTAAGCTCGAGTCACTTTGATGCTAGAGATTGTC

ZRANB3 into pFASTBac-His6-TEV
TACTTCCAATCCATGCCTAGGGTTCATAACATAA and TATCCACCTTTACTGTCACTTCTTTACCAAAAATCG

Introducing deletions into the HNH domain

ZRANB3-Δ975-1013
GTAATGTGAACGCACAAGAAGGACATTTCTGGCAGGTG and CACCTGCCAGAAATGTCCTTCTTGTGCGTTCACATTAC
ZRANB3-Δ972-1010
CAGCTCTGTAATGTGAACCCAGGGGAAGGACATTTC and GAAATGTCCTTCCCCTGGGTTCACATTACAGAGCTG

Mutation of the HNH domain

K984A
ACGTCTGAGAGATGCCCCTGCAAGTCAGAGGAAGAATCTT and AAGATTCTTCCTCTGACTTGCAGGGGCATCTCTCAGACGT
R987A
GAGATGCCCCTAAAAGTCAGGCGAAGAATCTTCTGTATGCTA and TAGCATACAGAAGATTCTTCGCCTGACTTTTAGGGGCATCTC
K988A
TGCCCCTAAAAGTCAGAGGGCGAATCTTCTGTATGCTACC and GGTAGCATACAGAAGATTCGCCCTCTGACTTTTAGGGGCA
K998A
GTATGCTACCTGGACTTCAGCGCTCCCATTAGAACAGCTAA and TTAGCTGTTCTAATGGGAGCGCTGAAGTCCAGGTAGCATA
R1009A
CCCATTAGAACAGCTAAATGAAATGATAGCAAACCCAGGGGAAGGA and TCCTTCCCCTGGGTTTGCTATCATTTCATTTAGCTGTTCTAATGGG
K1046A, R1048A
CTCTCTGCACAGTCTGTCACGCAGAGGCAACTGCCAGACAAGCTAAGG and
CCTTAGCTTGTCTGGCAGTTGCCTCTGCGTGACAGACTGTGCAGAGAG
H1015A
GAAACCCAGGGGAAGGAGCTTTCTGGCAGGTGGATC and GATCCACCTGCCAGAAAGCTCCTTCCCCTGGGTTTC
D1020A
CATTTCTGGCAGGTGGCTCACATCAAGCCAGTG and CACTGGCTTGATGTGAGCCACCTGCCAGAAATG
H1021A
CATTTCTGGCAGGTGGATGCCATCAAGCCAGTGTATGG and CCATACACTGGCTTGATGGCATCCACCTGCCAGAAATG
N1036A
GCAGAGAGTCTGCAGGGCGTCCAGGGAACACTGT and ACAGTGTTCCCTGGACGCCCTGCAGACTCTCTGC
H1045A
ACTCTCTGCACAGTCTGTGCCAAAGAGAGAACTGCCAG and CTGGCAGTTCTCTCTTTGGCACAGACTGTGCAGAGAGT

Mutation of the ZRANB3 PCNA binding motifs

Q519A
CACTCACTTCGAAAAAGAAAAAGCTCATGATATTCGATCATTTTTTG and CAAAAAATGATCGAATATCATGAGCTTTTTCTTTTTCGAAGTGAGTG
Q519A, F525A, F526A
GAAAAAGCTCATGATATTCGATCAGCTGCTGTACCACAACCTAAAAAAAGACAG and
GAAAAAGCTCATGATATTCGATCAGCTGCTGTACCACAACCTAAAAAAAGACAG
ΔAPIM:
TACTTCCAATCCATGCCTAGGGTTCATAACATAA and TATCCACCTTTACTGTTATGTGATGTCTGATCCATGCTTTG

PCNA into pET28a

Untagged
ATTGAGCTCATGATGTTCGAGGCGCGCC and ACTAACCTCGAGCTAAGATCCTTCTTCATCCTC
His-tagged
ACTAACCATATGTTCGAGGCGCGCC and ACTAACCTCGAGCTAAGATCCTTCTTCATCCTC

FEN1 into YFP
GGGGACAAGTTTGTACAAAAAAGCAGGCTTCATGGGAATTCAAGGCCTGGC and
GGGGACCACTTTGTACAAGAAAGCTGGGTATTATTTTCCCCTTTTAAACTTCCCTG

Mutation of the FEN1 PIP box

Q337A
CCAAGGCAGCACCGCGGGCCGCCTGGAT and ATCCAGGCGGCCCGCGGTGCTGCCTTGG
F343A, F344A
AGGGCCGCCTGGATGATGCCGCCAAGGTGACCGGCTCAC and GTGAGCCGGTCACCTTGGCGGCATCATCCAGGCGGCCCT

PCNA binding motifs into YFP

PIP
GGGGACAAGTTTGTACAAAAAAGCAGGCTTCATGCGAGCCCCTAAGAAAAAGCGGAAGGTGGGCGGCTTCACTCACTTCGAAAAAGAAAAACAG and GGGGACCACTTTGTACAAGAAAGCTGGGTATCATTTTTTAGGTTGTGGTACAAAAAATG

PIP*
GGGGACAAGTTTGTACAAAAAAGCAGGCTTCATGCGAGCCCCTAAGAAAAAGCGGAAGGTGGGCGGCTTCACTCACTTCGAAAAAGAAAAAGCT and GGGGACCACTTTGTACAAGAAAGCTGGGTATCATTTTTTAGGTTGTGGTACAGCAGCTG

APIM
GGGGACAAGTTTGTACAAAAAAGCAGGCTTCATGCGAGCCCCTAAGAAAAAGCGGAAGGTGGGCGGCCAGGTGAGAAGACAATCTCTAG and GGGGACCACTTTGTACAAGAAAGCTGGGTATTACTTCTTTACCAAAAATCGTGTGAT

APIM*
GGGGACAAGTTTGTACAAAAAAGCAGGCTTCATGCGAGCCCCTAAGAAAAAGCGGAAGGTGGGCGGCCAGGTGAGAAGACAATCTCTAG and GGGGACCACTTTGTACAAGAAAGCTGGGTATTACTTCTTTACCAAAGCTCGTGTGAT

ZRANB3 into YFP

WT
GGGGACAAGTTTGTACAAAAAAGCAGGCTTCATGCCTAGGGTTCATAACATAAAAAAG and GGGGACCACTTTGTACAAGAAAGCTGGGTATTACTTCTTTACCAAAAATCGTGTGATG

ΔAPIM
GGGGACAAGTTTGTACAAAAAAGCAGGCTTCATGCCTAGGGTTCATAACATAAAAAAG and GGGGACCACTTTGTACAAGAAAGCTGGGTATTATGTGATGTCTGATCCATGCTTTG

Cancer related ZRANB3 mutants

T66A
GAAATGGGTCTAGGAAAGGCAATCCAGGCAATTGGAA and TTCCAATTGCCTGGATTGCCTTTCCTAGACCCATTTC

R169H
GAAATCCAGAAATGCAACTCACAGCAGGATTTTATTGCCAA and TTGGCAATAAAATCCTGCTGTGAGTTGCATTTCTGGATTTC

G401D
TAAGCATTCAGGCTGCTGACCAGGGATTAACATTTAC and GTAAATGTTAATCCCTGGTCAGCAGCCTGAATGCTTA

R947Q
GTCAGGAAGAGTTTTGGATTCAATCTAATAACAGTTACCTGAG and CTCAGGTAACTGTTATTAGATTGAATCCAAAACTCTTCCTGAC

R947*
TGTCAGGAAGAGTTTTGGATTTGATCTAATAACAGTTACCTG and CAGGTAACTGTTATTAGATCAAATCCAAAACTCTTCCTGACA

D1020Y
GACATTTCTGGCAGGTGTATCACATCAAGCCAGTG and CACTGGCTTGATGTGATACACCTGCCAGAAATGTC
